# Supplementary material for: Systemic viral spreading and defective host responses are associated with fatal Lassa fever in macaques
Source: Commun Biol. 2021 Jan 4;4:27. doi: 10.1038/s42003-020-01543-7 (PMC7782745; doi:10.1038/s42003-020-01543-7)
Supplement: Supplementary file 2 — Supplementary Information [file 42003_2020_1543_MOESM2_ESM.pdf]

Supplementary Information for

Systemic viral spreading and defective host responses are associated with fatal Lassa fever in macaques

Nicolas Baillet[1,2], Stéphanie Reynard[1,2], Emeline Perthame[3], Jimmy Hortion[1,2], Alexandra Journeaux[1,2], Mathieu Mateo[1,2], Xavier Carnec[1,2], Justine Schaeffer[1,2], Caroline Picard[1,2], Laura Barrot[4], Stéphane Barron[4], Audrey Vallve[4], Aurélie Duthey[4], Frédéric Jacquot[4], Cathy Boehringer[4], Grégory Jouvion[5], Natalia Pietrosemoli[3], Rachel Legendre[3], Marie-Agnès Dillies[3], Richard Allan[6], Catherine Legras-Lachuer[6], Caroline Carbonnelle[4], Hervé Raoul[4], and Sylvain Baize[1,2*]

[1]Unité de Biologie des Infections Virales Emergentes, Institut Pasteur, Lyon, France

[2]Centre International de Recherche en Infectiologie (CIRI), Université de Lyon, INSERM U1111, Ecole Normale Supérieure de Lyon, Université Lyon 1, CNRS UMR5308, Lyon, France

[3]Hub de Bioinformatique et Biostatistique – Département Biologie Computationnelle, Institut Pasteur, USR 3756 CNRS, Paris, France

[4]Laboratoire P4 INSERM – Jean Mérieux, INSERM US003, Lyon, France

[5]Neuropathologie Expérimentale, Département de Santé Globale, Institut Pasteur, Paris, France

[6]ViroScan3D SAS, Trévoux, France

Supplemental Figures

Fig. S1. Supplementary clinical and virological features after LASV challenge 2

Fig. S2. Anatomopathological changes of organs after LASV challenge 4

Fig. S3. Analysis of memory T-cell responses 6

Fig S4. Heatmaps of differentially expressed genes for the PBMC dataset 8

Fig S5. Venn diagrams 10

Fig S6. Heatmaps of the most significant pathways by gene-set analysis in PBMCs 11

Fig S7. Heatmaps of the most significant pathways by gene-set analysis in the organs 14

Fig S1

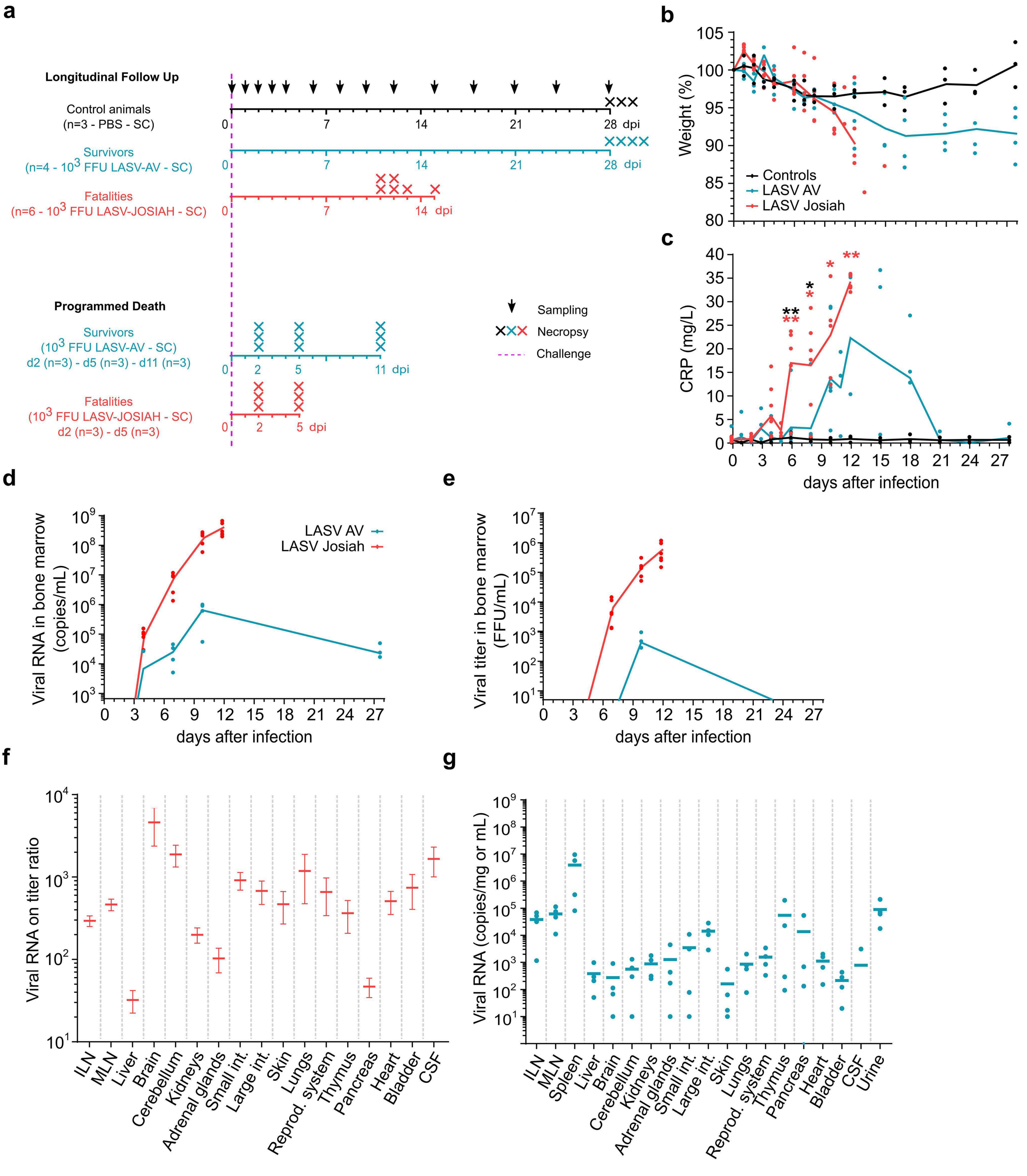

a
Longitudinal Follow Up
Control animals
(n=3 - PBS - SC)
Survivors
(n=4 - 10^3 FFU LASV-AV - SC)
Fatalities
(n=6 - 10^3 FFU LASV-JOSIAH - SC)
Programmed Death
Survivors
(10^3 FFU LASV-AV - SC)
d2 (n=3) - d5 (n=3) - d11 (n=3)
Fatalities
(10^3 FFU LASV-JOSIAH - SC)
d2 (n=3) - d5 (n=3)
dpi
Sampling
Necropsy
Challenge
b
Weight (%)
Controls
LASV AV
LASV Josiah
c
CRP (mg/L)
days after infection
d
Viral RNA in bone marrow (copies/mL)
LASV AV
LASV Josiah
days after infection
e
Viral titer in bone marrow (FFU/mL)
days after infection
f
Viral RNA on titer ratio
ILN
MLN
Liver
Brain
Cerebellum
Kidneys
Adrenal glands
Small int.
Large int.
Skin
Lungs
Reprod. system
Thymus
Pancreas
Heart
Bladder
CSF
g
Viral RNA (copies/mg or mL)
ILN
MLN
Spleen
Liver
Brain
Cerebellum
Kidneys
Adrenal glands
Small int.
Large int.
Skin
Lungs
Reprod. system
Thymus
Pancreas
Heart
Bladder
CSF
Urine

Fig. S1. Supplementary clinical and virological features after LASV challenge. **a**. Scheme presenting the time of challenge, sampling, and necropsy for each group. SC: Subcutaneous; FFU: focus-forming unit. **b**. Monitoring of weight during the course of infection. Individual data (points) and mean values for each cohort (curves) are presented. Controls (n = 3), LASV-AV (n = 4), LASV-Josiah (n = 6). **c**. Analysis of CRP levels in the plasma of animals during LASV infection. Results show the mean and individual values for each group. Controls (n = 3), LASV-AV (n = 4), LASV-Josiah (n = 6). Statistical analyses were performed and are presented as in Fig. 1. **d.** Quantification of viral load by RT-qPCR in the BM of animals. Mean values of each cohort are presented as the number of viral RNA copies/ml according to the time after challenge. **e.** Quantification of LASV infectious particles in BM according to the time after challenge in FFU/ml. **(d, e)** LASV-AV (n = 4), LASV-Josiah (n = 6). **f**. Viral RNA to titer ratio calculated from the organ samples of LASV-Josiah infected animals (n = 6) at approximately 12 DPI. Results show the mean ± SEM of the ratio. **g**. RT-qPCR quantification of viral RNA copies/mg of various tissues from four LASV-AV infected animals 28 DPI. Individual and mean values are presented.

Fig S2

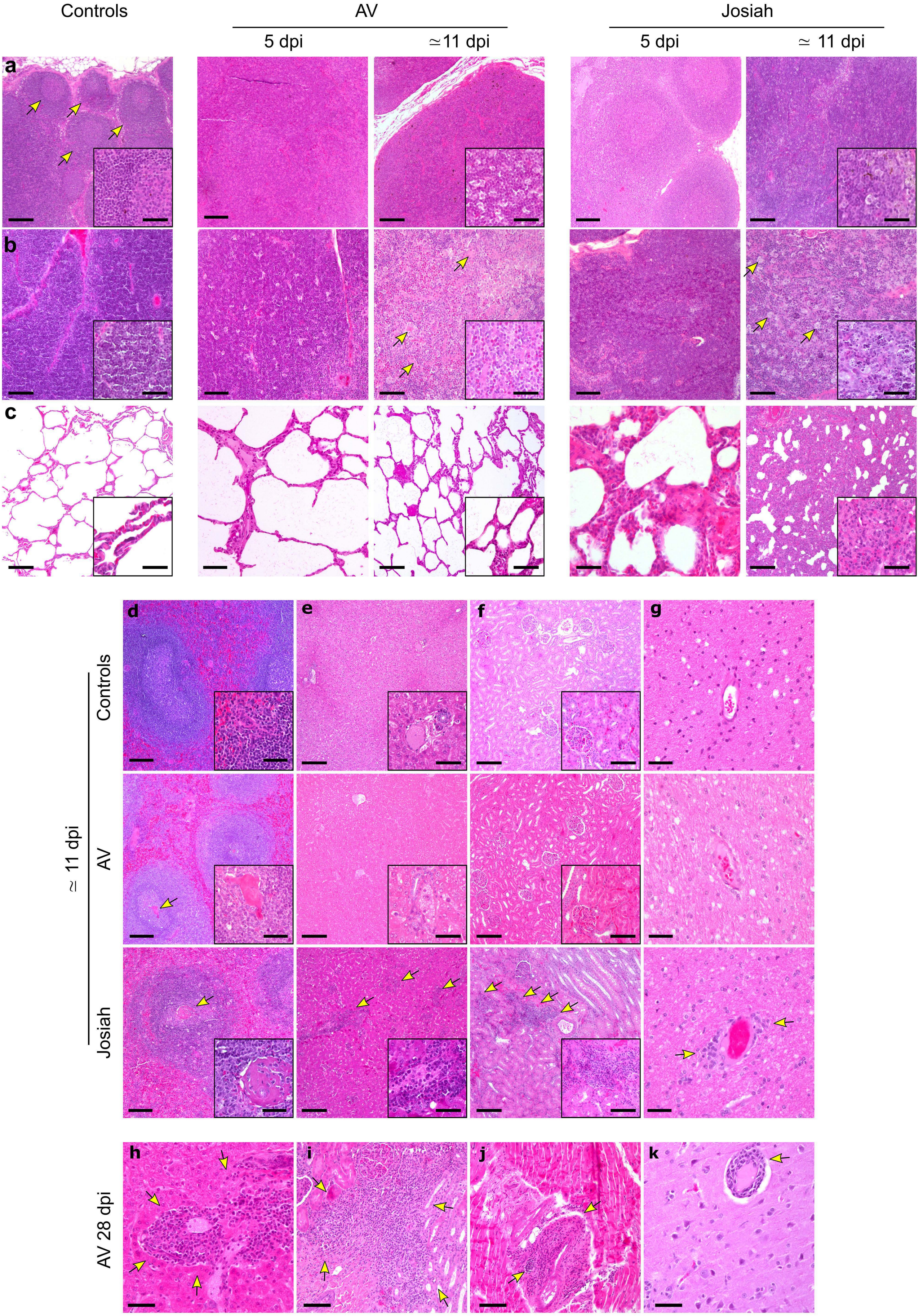

Controls
AV
Josiah
5 dpi
≃11 dpi
5 dpi
≃ 11 dpi
a
b
c
d
e
f
g
Controls
AV
Josiah
≃ 11 dpi
h
i
j
k
AV 28 dpi

Fig. S2. Anatomopathological changes of organs after LASV challenge. Hematoxylin and eosin slides of **(a)** lymph node, **(b)** thymus, **(c)** lungs, **(d)** spleen, **(e)** liver, **(f)** kidneys, and **(g)** brain at 5 and 11 DPI in infected primates. Hematoxylin and eosin slides of **(h)** liver, **(i)** kidneys, **(j)** cardiac muscle, and **(l)** brain at 28 DPI in LASV-AV infected primates. **(a-e)**, Scale bars: 250 µm; Scale bars for the higher magnification images in the boxes in the lower-right corner: 50 µm. **(f)** Scale bars: 250 µm; Scale bars for the higher magnification images in the boxes in the lower-right corner: 100 µm. **(g-i)** Scale bars: 100 µm**. (j,k)** Scale bars: 200 µm. Key anatomopathological features are indicated by arrows.

Fig S3

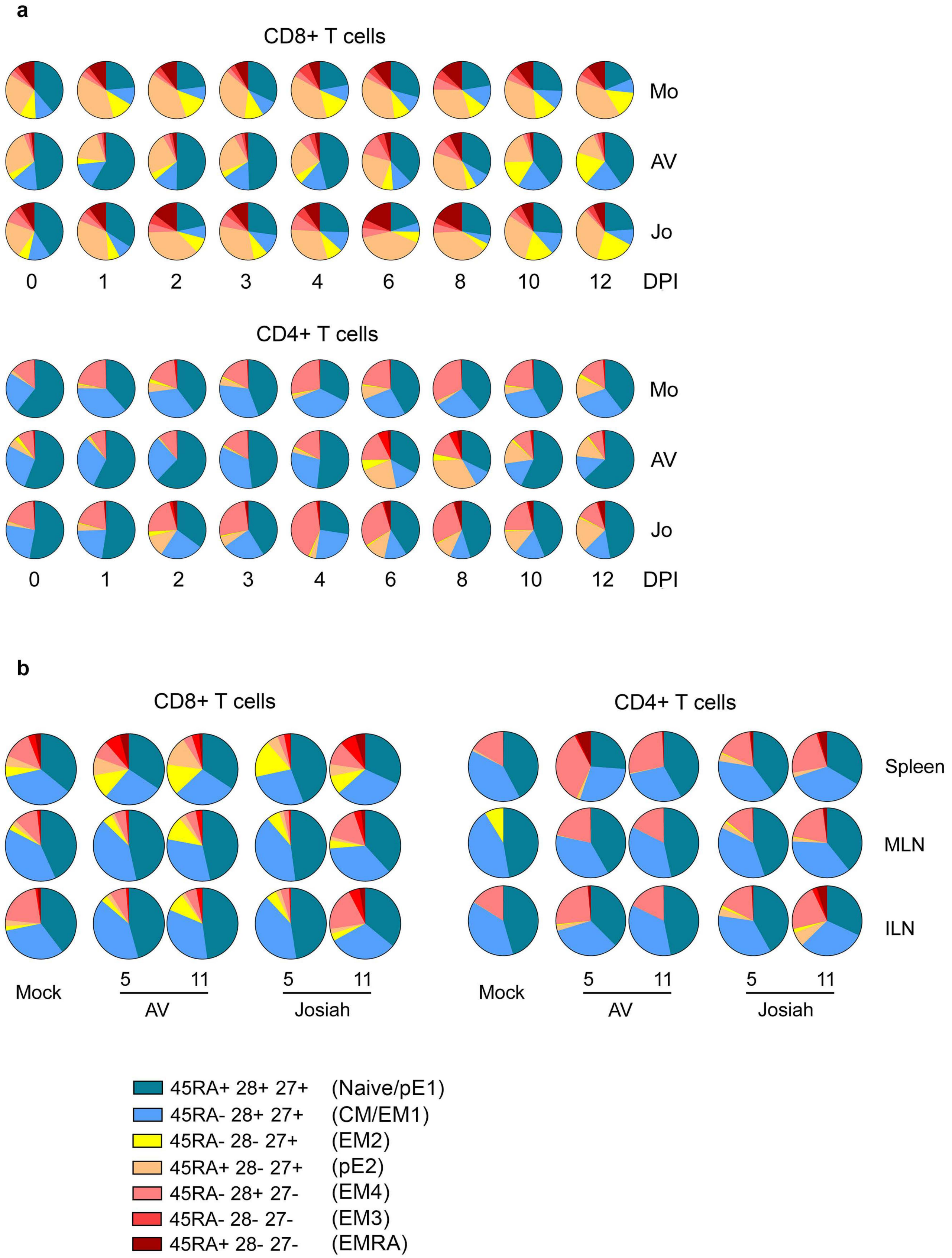
a
CD8+ T cells
Mo
AV
Jo
0 1 2 3 4 6 8 10 12 DPI
CD4+ T cells
Mo
AV
Jo
0 1 2 3 4 6 8 10 12 DPI
b
CD8+ T cells
CD4+ T cells
Spleen
MLN
ILN
Mock
5 11
AV
5 11
Josiah
Mock
5 11
AV
5 11
Josiah
45RA+ 28+ 27+ (Naive/pE1)
45RA- 28+ 27+ (CM/EM1)
45RA- 28- 27+ (EM2)
45RA+ 28- 27+ (pE2)
45RA- 28+ 27- (EM4)
45RA- 28- 27- (EM3)
45RA+ 28- 27- (EMRA)

Fig. S3. Analysis of memory T-cell responses. **a**. The proportion of memory $CD8^+$ and $CD4^+$ T-cell subpopulations in the blood according to CD45RA, CD28, and CD27 expression is presented according to the time after infection. The subpopulations are naïve, pre-effector 1/2 (pE1/2), central memory (CM), effector memory 1/2/3/4 (EM1/2/3/4), and terminally differentiated effector memory (EMRA) T cells. Results show the mean of the data for each group: Controls (n = 3), LASV-AV (n = 3), LASV-Josiah (n = 6). **b**. The proportion of memory $CD8^+$ and $CD4^+$ T-cell subpopulations in the spleen, MLNs, and ILNs according to CD45RA, CD28, and CD27 expression is presented according to the time after infection. Results show the mean of the data for each group: Controls (n = 3), LASV-AV (n = 3), LASV-Josiah (n = 6). No statistical analysis was performed for these data.

Fig S4

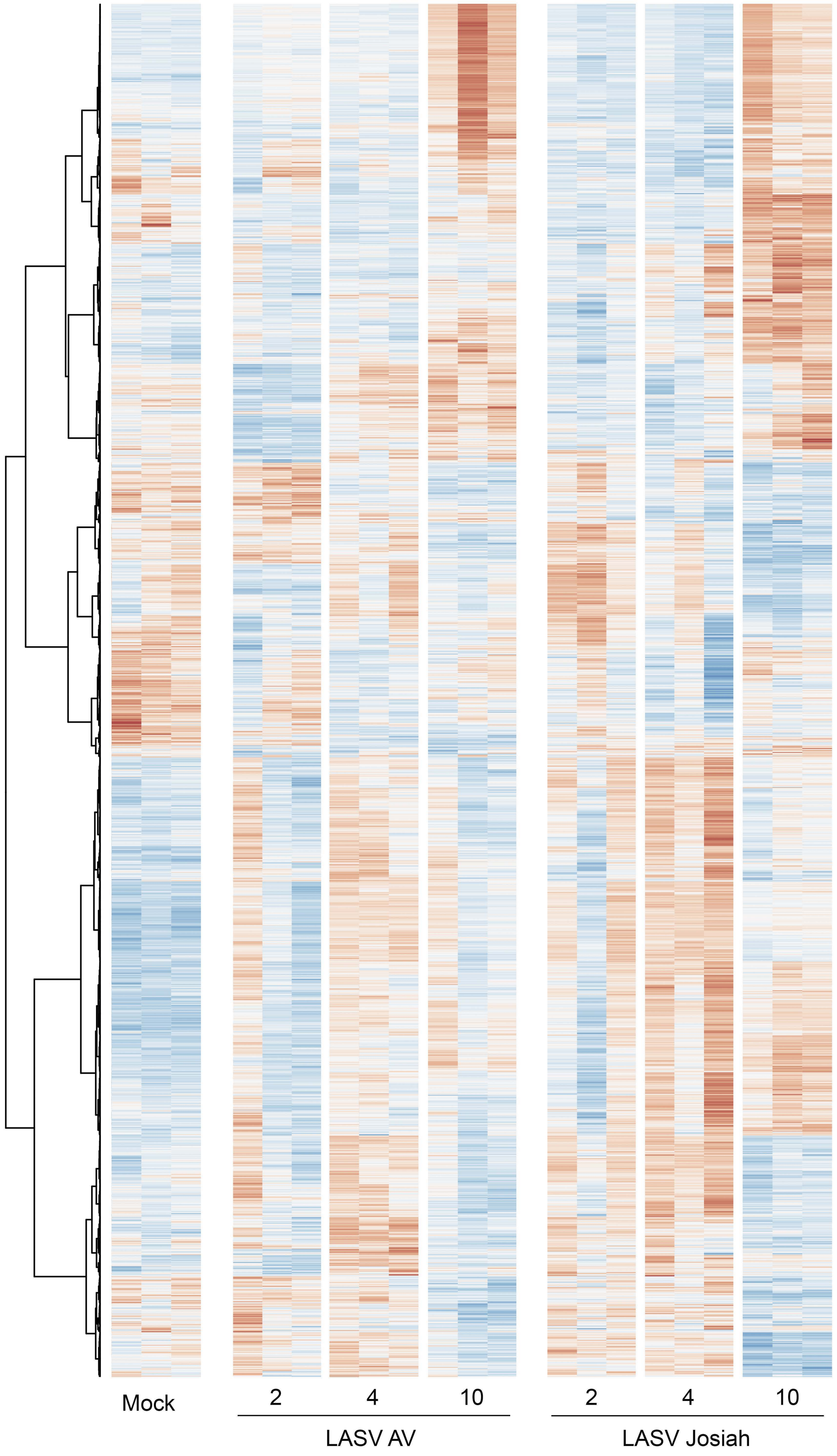

Fig S4. Heatmaps of differentially expressed genes for the PBMC dataset. Gene expression was standardized using VST transformation, hence centered and scaled to make the gene expression comparable. This heatmap displays gene expression in PBMCs for each individual at 2, 4, and 10 DPI, as well as in PBMCs from mock animals.

Fig S5

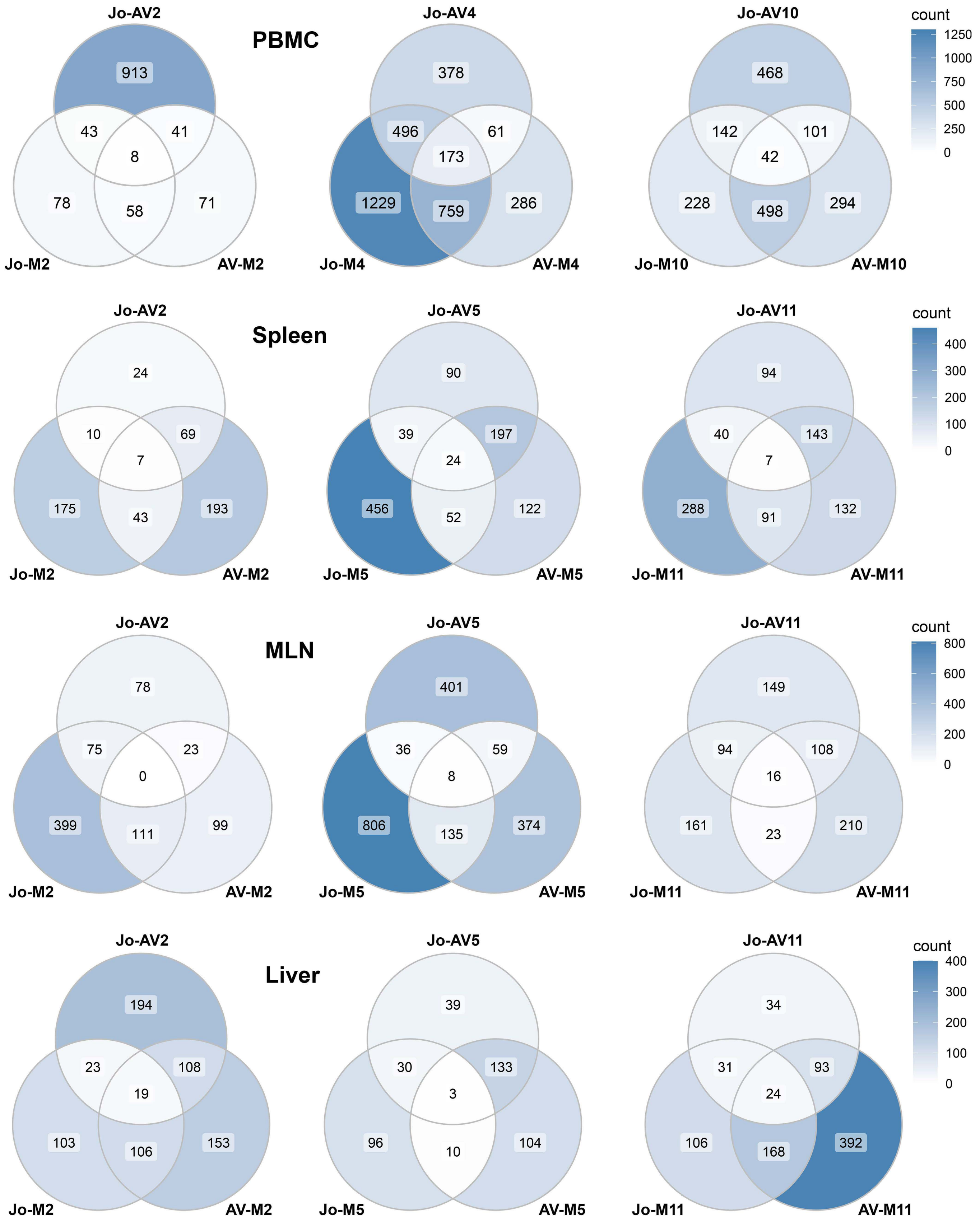

Fig S5. Venn diagrams. Venn diagrams showing the numbers of differentially expressed genes and overlaps per timepoint among the comparisons for Josiah- (Jo), AV-, and Mock- (M) infected animals in PBMC and organs. The label outside each circle indicates the time and the groups to which the comparison applied. The color scale indicates the number of DE genes for each comparison.

Fig S6a

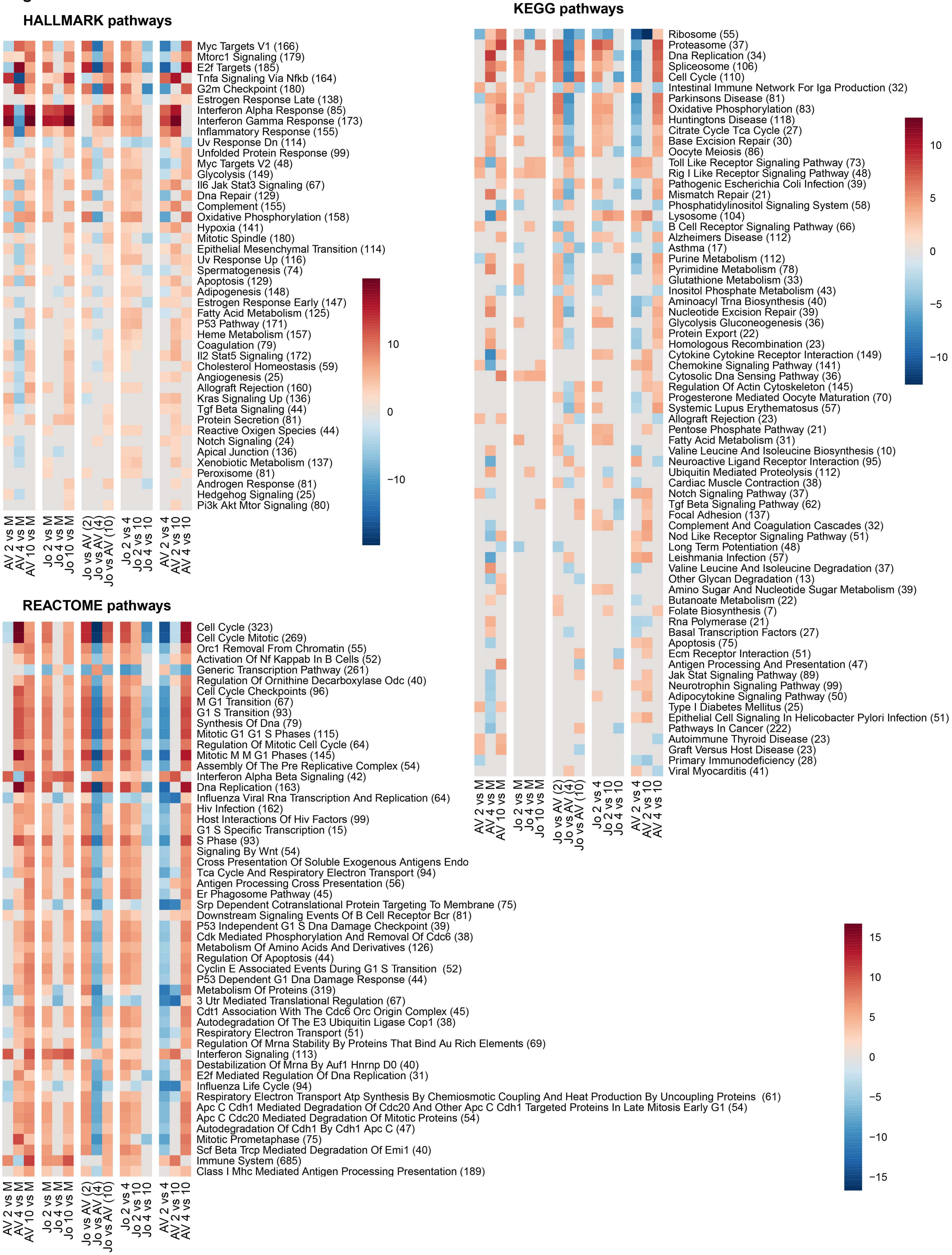
HALLMARK pathways
Myc Targets V1 (166)
Mtorc1 Signaling (179)
E2f Targets (185)
Tnfa Signaling Via Nfkb (164)
G2m Checkpoint (180)
Estrogen Response Late (138)
Interferon Alpha Response (85)
Interferon Gamma Response (173)
Inflammatory Response (155)
Uv Response Dn (114)
Unfolded Protein Response (99)
Myc Targets V2 (48)
Glycolysis (149)
Il6 Jak Stat3 Signaling (67)
Dna Repair (129)
Complement (155)
Oxidative Phosphorylation (158)
Hypoxia (141)
Mitotic Spindle (180)
Epithelial Mesenchymal Transition (114)
Uv Response Up (116)
Spermatogenesis (74)
Apoptosis (129)
Adipogenesis (148)
Estrogen Response Early (147)
Fatty Acid Metabolism (125)
P53 Pathway (171)
Heme Metabolism (157)
Coagulation (79)
Il2 Stat5 Signaling (172)
Cholesterol Homeostasis (59)
Angiogenesis (25)
Allograft Rejection (160)
Kras Signaling Up (136)
Tgf Beta Signaling (44)
Protein Secretion (81)
Reactive Oxigen Species (44)
Notch Signaling (24)
Apical Junction (136)
Xenobiotic Metabolism (137)
Peroxisome (81)
Androgen Response (81)
Hedgehog Signaling (25)
Pi3k Akt Mtor Signaling (80)
10
0
−10
AV 2 vs M
AV 4 vs M
AV 10 vs M
Jo 2 vs M
Jo 4 vs M
Jo 10 vs M
Jo vs AV (2)
Jo vs AV (4)
Jo vs AV (10)
Jo 2 vs 4
Jo 2 vs 10
Jo 4 vs 10
AV 2 vs 4
AV 2 vs 10
AV 4 vs 10
KEGG pathways
Ribosome (55)
Proteasome (37)
Dna Replication (34)
Spliceosome (106)
Cell Cycle (110)
Intestinal Immune Network For Iga Production (32)
Parkinsons Disease (81)
Oxidative Phosphorylation (83)
Huntingtons Disease (118)
Citrate Cycle Tca Cycle (27)
Base Excision Repair (30)
Oocyte Meiosis (86)
Toll Like Receptor Signaling Pathway (73)
Rig I Like Receptor Signaling Pathway (48)
Pathogenic Escherichia Coli Infection (39)
Mismatch Repair (21)
Phosphatidylinositol Signaling System (58)
Lysosome (104)
B Cell Receptor Signaling Pathway (66)
Alzheimers Disease (112)
Asthma (17)
Purine Metabolism (112)
Pyrimidine Metabolism (78)
Glutathione Metabolism (33)
Inositol Phosphate Metabolism (43)
Aminoacyl Trna Biosynthesis (40)
Nucleotide Excision Repair (39)
Glycolysis Gluconeogenesis (36)
Protein Export (22)
Homologous Recombination (23)
Cytokine Cytokine Receptor Interaction (149)
Chemokine Signaling Pathway (141)
Cytosolic Dna Sensing Pathway (36)
Regulation Of Actin Cytoskeleton (145)
Progesterone Mediated Oocyte Maturation (70)
Systemic Lupus Erythematosus (57)
Allograft Rejection (23)
Pentose Phosphate Pathway (21)
Fatty Acid Metabolism (31)
Valine Leucine And Isoleucine Biosynthesis (10)
Neuroactive Ligand Receptor Interaction (95)
Ubiquitin Mediated Proteolysis (112)
Cardiac Muscle Contraction (38)
Notch Signaling Pathway (37)
Tgf Beta Signaling Pathway (62)
Focal Adhesion (137)
Complement And Coagulation Cascades (32)
Nod Like Receptor Signaling Pathway (51)
Long Term Potentiation (48)
Leishmania Infection (57)
Valine Leucine And Isoleucine Degradation (37)
Other Glycan Degradation (13)
Amino Sugar And Nucleotide Sugar Metabolism (39)
Butanoate Metabolism (22)
Folate Biosynthesis (7)
Rna Polymerase (21)
Basal Transcription Factors (27)
Apoptosis (75)
Ecm Receptor Interaction (51)
Antigen Processing And Presentation (47)
Jak Stat Signaling Pathway (89)
Neurotrophin Signaling Pathway (99)
Adipocytokine Signaling Pathway (50)
Type I Diabetes Mellitus (25)
Epithelial Cell Signaling In Helicobacter Pylori Infection (51)
Pathways In Cancer (222)
Autoimmune Thyroid Disease (23)
Graft Versus Host Disease (23)
Primary Immunodeficiency (28)
Viral Myocarditis (41)
10
5
0
−5
−10
AV 2 vs M
AV 4 vs M
AV 10 vs M
Jo 2 vs M
Jo 4 vs M
Jo 10 vs M
Jo vs AV (2)
Jo vs AV (4)
Jo vs AV (10)
Jo 2 vs 4
Jo 2 vs 10
Jo 4 vs 10
AV 2 vs 4
AV 2 vs 10
AV 4 vs 10
REACTOME pathways
Cell Cycle (323)
Cell Cycle Mitotic (269)
Orc1 Removal From Chromatin (55)
Activation Of Nf Kappab In B Cells (52)
Generic Transcription Pathway (261)
Regulation Of Ornithine Decarboxylase Odc (40)
Cell Cycle Checkpoints (96)
M G1 Transition (67)
G1 S Transition (93)
Synthesis Of Dna (79)
Mitotic G1 G1 S Phases (115)
Regulation Of Mitotic Cell Cycle (64)
Mitotic M M G1 Phases (145)
Assembly Of The Pre Replicative Complex (54)
Interferon Alpha Beta Signaling (42)
Dna Replication (163)
Influenza Viral Rna Transcription And Replication (64)
Hiv Infection (162)
Host Interactions Of Hiv Factors (99)
G1 S Specific Transcription (15)
S Phase (93)
Signaling By Wnt (54)
Cross Presentation Of Soluble Exogenous Antigens Endo
Tca Cycle And Respiratory Electron Transport (94)
Antigen Processing Cross Presentation (56)
Er Phagosome Pathway (45)
Srp Dependent Cotranslational Protein Targeting To Membrane (75)
Downstream Signaling Events Of B Cell Receptor Bcr (81)
P53 Independent G1 S Dna Damage Checkpoint (39)
Cdk Mediated Phosphorylation And Removal Of Cdc6 (38)
Metabolism Of Amino Acids And Derivatives (126)
Regulation Of Apoptosis (44)
Cyclin E Associated Events During G1 S Transition (52)
P53 Dependent G1 Dna Damage Response (44)
Metabolism Of Proteins (319)
3 Utr Mediated Translational Regulation (67)
Cdt1 Association With The Cdc6 Orc Origin Complex (45)
Autodegradation Of The E3 Ubiquitin Ligase Cop1 (38)
Respiratory Electron Transport (51)
Regulation Of Mrna Stability By Proteins That Bind Au Rich Elements (69)
Interferon Signaling (113)
Destabilization Of Mrna By Auf1 Hnrnp D0 (40)
E2f Mediated Regulation Of Dna Replication (31)
Influenza Life Cycle (94)
Respiratory Electron Transport Atp Synthesis By Chemiosmotic Coupling And Heat Production By Uncoupling Proteins (61)
Apc C Cdh1 Mediated Degradation Of Cdc20 And Other Apc C Cdh1 Targeted Proteins In Late Mitosis Early G1 (54)
Apc C Cdc20 Mediated Degradation Of Mitotic Proteins (54)
Autodegradation Of Cdh1 By Cdh1 Apc C (47)
Mitotic Prometaphase (75)
Scf Beta Trcp Mediated Degradation Of Emi1 (40)
Immune System (685)
Class I Mhc Mediated Antigen Processing Presentation (189)
15
10
5
0
−5
−10
−15
AV 2 vs M
AV 4 vs M
AV 10 vs M
Jo 2 vs M
Jo 4 vs M
Jo 10 vs M
Jo vs AV (2)
Jo vs AV (4)
Jo vs AV (10)
Jo 2 vs 4
Jo 2 vs 10
Jo 4 vs 10
AV 2 vs 4
AV 2 vs 10
AV 4 vs 10

Fig S6b

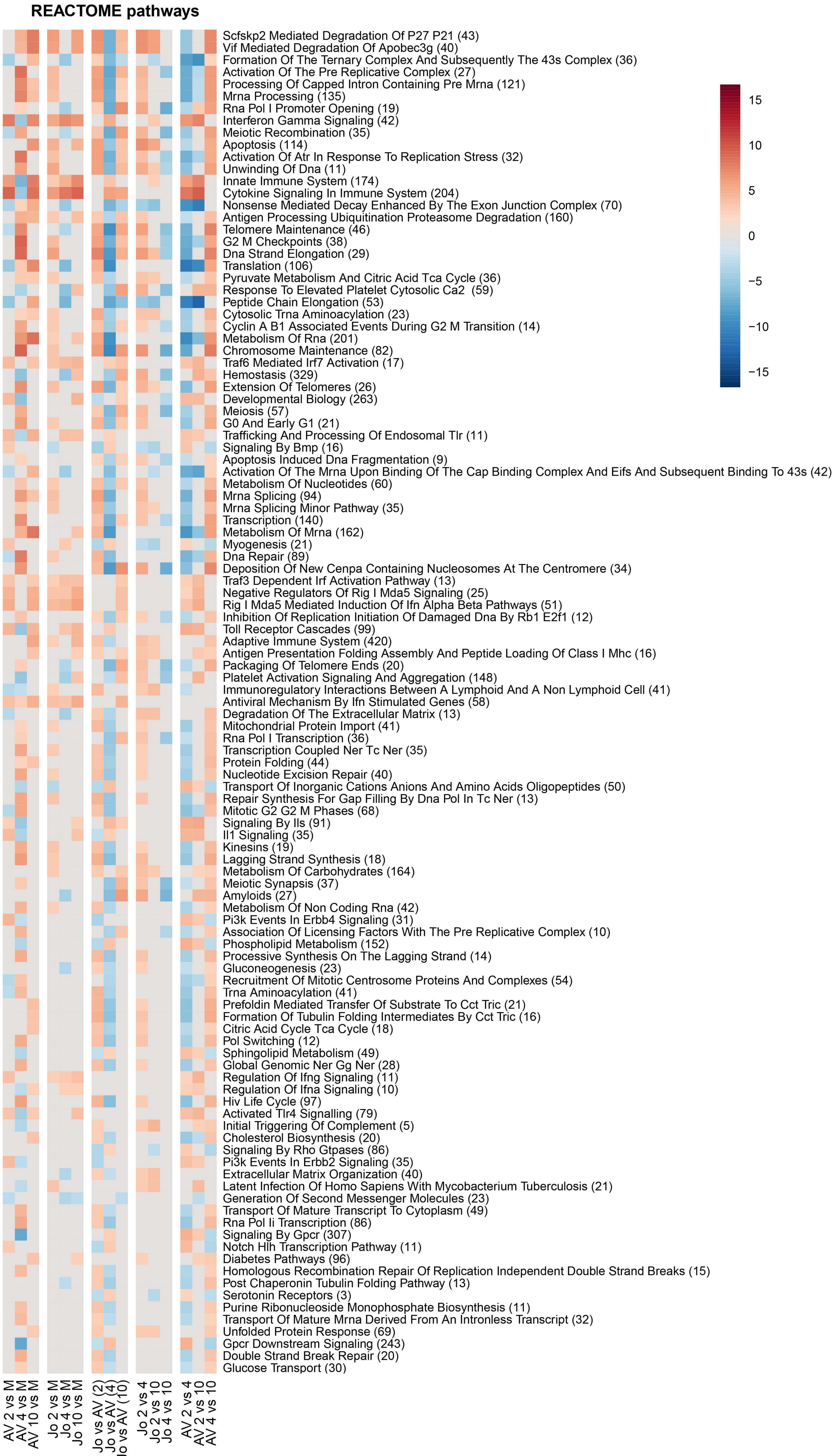
REACTOME pathways
Scfskp2 Mediated Degradation Of P27 P21 (43)
Vif Mediated Degradation Of Apobec3g (40)
Formation Of The Ternary Complex And Subsequently The 43s Complex (36)
Activation Of The Pre Replicative Complex (27)
Processing Of Capped Intron Containing Pre Mrna (121)
Mrna Processing (135)
Rna Pol I Promoter Opening (19)
Interferon Gamma Signaling (42)
Meiotic Recombination (35)
Apoptosis (114)
Activation Of Atr In Response To Replication Stress (32)
Unwinding Of Dna (11)
Innate Immune System (174)
Cytokine Signaling In Immune System (204)
Nonsense Mediated Decay Enhanced By The Exon Junction Complex (70)
Antigen Processing Ubiquitination Proteasome Degradation (160)
Telomere Maintenance (46)
G2 M Checkpoints (38)
Dna Strand Elongation (29)
Translation (106)
Pyruvate Metabolism And Citric Acid Tca Cycle (36)
Response To Elevated Platelet Cytosolic Ca2 (59)
Peptide Chain Elongation (53)
Cytosolic Trna Aminoacylation (23)
Cyclin A B1 Associated Events During G2 M Transition (14)
Metabolism Of Rna (201)
Chromosome Maintenance (82)
Traf6 Mediated Irf7 Activation (17)
Hemostasis (329)
Extension Of Telomeres (26)
Developmental Biology (263)
Meiosis (57)
G0 And Early G1 (21)
Trafficking And Processing Of Endosomal Tlr (11)
Signaling By Bmp (16)
Apoptosis Induced Dna Fragmentation (9)
Activation Of The Mrna Upon Binding Of The Cap Binding Complex And Eifs And Subsequent Binding To 43s (42)
Metabolism Of Nucleotides (60)
Mrna Splicing (94)
Mrna Splicing Minor Pathway (35)
Transcription (140)
Metabolism Of Mrna (162)
Myogenesis (21)
Dna Repair (89)
Deposition Of New Cenpa Containing Nucleosomes At The Centromere (34)
Traf3 Dependent Irf Activation Pathway (13)
Negative Regulators Of Rig I Mda5 Signaling (25)
Rig I Mda5 Mediated Induction Of Ifn Alpha Beta Pathways (51)
Inhibition Of Replication Initiation Of Damaged Dna By Rb1 E2f1 (12)
Toll Receptor Cascades (99)
Adaptive Immune System (420)
Antigen Presentation Folding Assembly And Peptide Loading Of Class I Mhc (16)
Packaging Of Telomere Ends (20)
Platelet Activation Signaling And Aggregation (148)
Immunoregulatory Interactions Between A Lymphoid And A Non Lymphoid Cell (41)
Antiviral Mechanism By Ifn Stimulated Genes (58)
Degradation Of The Extracellular Matrix (13)
Mitochondrial Protein Import (41)
Rna Pol I Transcription (36)
Transcription Coupled Ner Tc Ner (35)
Protein Folding (44)
Nucleotide Excision Repair (40)
Transport Of Inorganic Cations Anions And Amino Acids Oligopeptides (50)
Repair Synthesis For Gap Filling By Dna Pol In Tc Ner (13)
Mitotic G2 G2 M Phases (68)
Signaling By Ils (91)
Il1 Signaling (35)
Kinesins (19)
Lagging Strand Synthesis (18)
Metabolism Of Carbohydrates (164)
Meiotic Synapsis (37)
Amyloids (27)
Metabolism Of Non Coding Rna (42)
Pi3k Events In Erbb4 Signaling (31)
Association Of Licensing Factors With The Pre Replicative Complex (10)
Phospholipid Metabolism (152)
Processive Synthesis On The Lagging Strand (14)
Gluconeogenesis (23)
Recruitment Of Mitotic Centrosome Proteins And Complexes (54)
Trna Aminoacylation (41)
Prefoldin Mediated Transfer Of Substrate To Cct Tric (21)
Formation Of Tubulin Folding Intermediates By Cct Tric (16)
Citric Acid Cycle Tca Cycle (18)
Pol Switching (12)
Sphingolipid Metabolism (49)
Global Genomic Ner Gg Ner (28)
Regulation Of Ifng Signaling (11)
Regulation Of Ifna Signaling (10)
Hiv Life Cycle (97)
Activated Tlr4 Signalling (79)
Initial Triggering Of Complement (5)
Cholesterol Biosynthesis (20)
Signaling By Rho Gtpases (86)
Pi3k Events In Erbb2 Signaling (35)
Extracellular Matrix Organization (40)
Latent Infection Of Homo Sapiens With Mycobacterium Tuberculosis (21)
Generation Of Second Messenger Molecules (23)
Transport Of Mature Transcript To Cytoplasm (49)
Rna Pol Ii Transcription (86)
Signaling By Gpcr (307)
Notch Hlh Transcription Pathway (11)
Diabetes Pathways (96)
Homologous Recombination Repair Of Replication Independent Double Strand Breaks (15)
Post Chaperonin Tubulin Folding Pathway (13)
Serotonin Receptors (3)
Purine Ribonucleoside Monophosphate Biosynthesis (11)
Transport Of Mature Mrna Derived From An Intronless Transcript (32)
Unfolded Protein Response (69)
Gpcr Downstream Signaling (243)
Double Strand Break Repair (20)
Glucose Transport (30)
15
10
5
0
−5
−10
−15
AV 2 vs M
AV 4 vs M
AV 10 vs M
Jo 2 vs M
Jo 4 vs M
Jo 10 vs M
Jo vs AV (2)
Jo vs AV (4)
Jo vs AV (10)
Jo 2 vs 4
Jo 2 vs 10
Jo 4 vs 10
AV 2 vs 4
AV 2 vs 10
AV 4 vs 10

Fig S6c

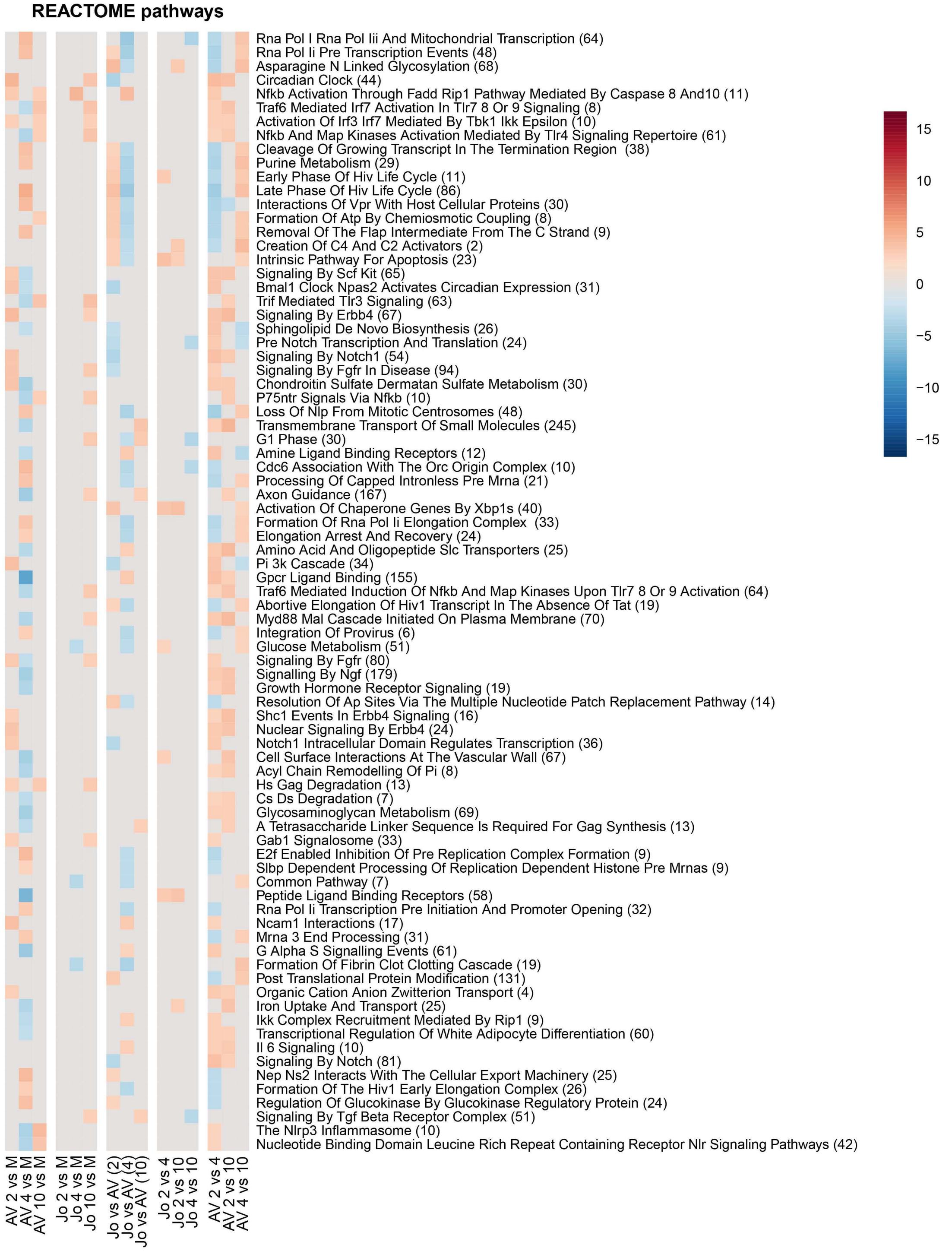

Fig S6. Heatmaps of the most significant pathways by gene-set analysis in PBMCs. Heatmaps of the CAMERA scores for the most significant pathways in gene-set analysis applied to the Hallmark, KEGG (Fig. S6a), and Reactome (Fig. S6a-c) gene sets (at least three significant comparisons) are presented for PBMCs. Comparisons between groups are shown on the x-axis and gene sets on the y-axis. The number of genes contained in each pathway is indicated in parenthesis. Grey squares indicate not-significantly regulated gene sets, whereas the red and blue squares indicate pathways that were significantly upregulated (red squares) or downregulated (blue squares) between the two groups. A gene set was considered to be downregulated if its score in CAMERA analysis was positive and downregulated if its score was negative.

Fig S7a

Liver

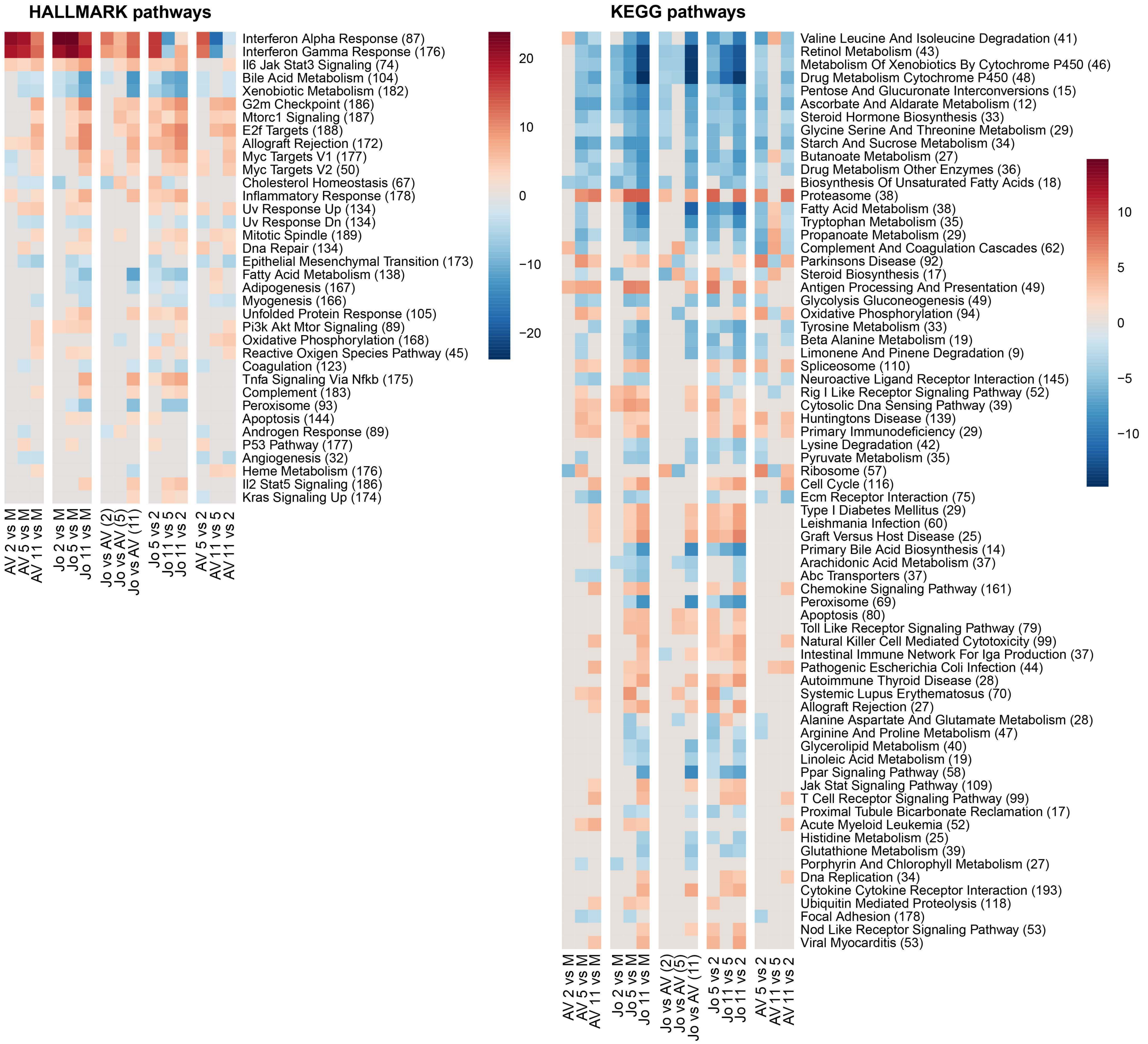

Fig S7b

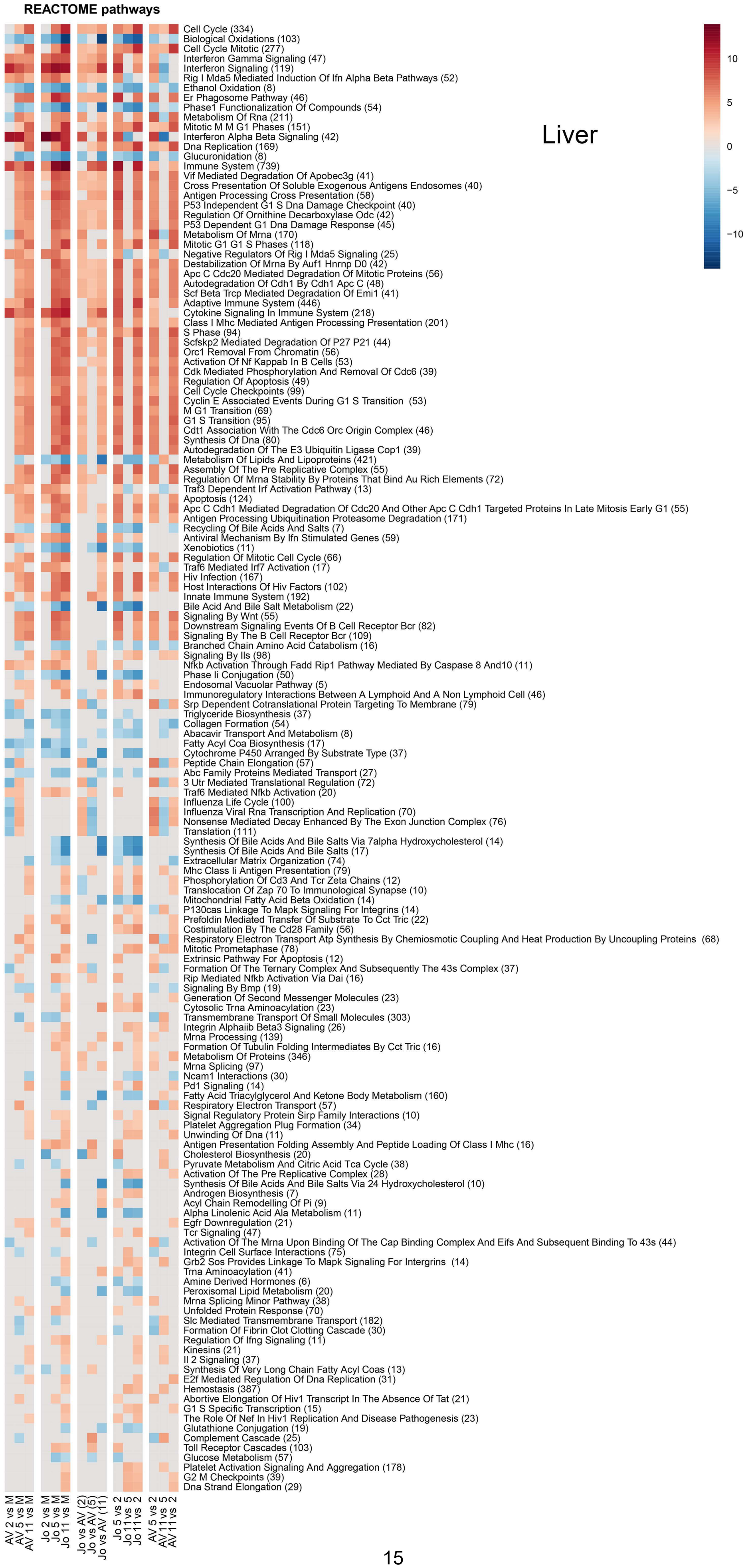
REACTOME pathways
Liver
10
5
0
−5
−10
Cell Cycle (334)
Biological Oxidations (103)
Cell Cycle Mitotic (277)
Interferon Gamma Signaling (47)
Interferon Signaling (119)
Rig I Mda5 Mediated Induction Of Ifn Alpha Beta Pathways (52)
Ethanol Oxidation (8)
Er Phagosome Pathway (46)
Phase1 Functionalization Of Compounds (54)
Metabolism Of Rna (211)
Mitotic M M G1 Phases (151)
Interferon Alpha Beta Signaling (42)
Dna Replication (169)
Glucuronidation (8)
Immune System (739)
Vif Mediated Degradation Of Apobec3g (41)
Cross Presentation Of Soluble Exogenous Antigens Endosomes (40)
Antigen Processing Cross Presentation (58)
P53 Independent G1 S Dna Damage Checkpoint (40)
Regulation Of Ornithine Decarboxylase Odc (42)
P53 Dependent G1 Dna Damage Response (45)
Metabolism Of Mrna (170)
Mitotic G1 G1 S Phases (118)
Negative Regulators Of Rig I Mda5 Signaling (25)
Destabilization Of Mrna By Auf1 Hnrnp D0 (42)
Apc C Cdc20 Mediated Degradation Of Mitotic Proteins (56)
Autodegradation Of Cdh1 By Cdh1 Apc C (48)
Scf Beta Trcp Mediated Degradation Of Emi1 (41)
Adaptive Immune System (446)
Cytokine Signaling In Immune System (218)
Class I Mhc Mediated Antigen Processing Presentation (201)
S Phase (94)
Scfskp2 Mediated Degradation Of P27 P21 (44)
Orc1 Removal From Chromatin (56)
Activation Of Nf Kappab In B Cells (53)
Cdk Mediated Phosphorylation And Removal Of Cdc6 (39)
Regulation Of Apoptosis (49)
Cell Cycle Checkpoints (99)
Cyclin E Associated Events During G1 S Transition (53)
M G1 Transition (69)
G1 S Transition (95)
Cdt1 Association With The Cdc6 Orc Origin Complex (46)
Synthesis Of Dna (80)
Autodegradation Of The E3 Ubiquitin Ligase Cop1 (39)
Metabolism Of Lipids And Lipoproteins (421)
Assembly Of The Pre Replicative Complex (55)
Regulation Of Mrna Stability By Proteins That Bind Au Rich Elements (72)
Traf3 Dependent Irf Activation Pathway (13)
Apoptosis (124)
Apc C Cdh1 Mediated Degradation Of Cdc20 And Other Apc C Cdh1 Targeted Proteins In Late Mitosis Early G1 (55)
Antigen Processing Ubiquitination Proteasome Degradation (171)
Recycling Of Bile Acids And Salts (7)
Antiviral Mechanism By Ifn Stimulated Genes (59)
Xenobiotics (11)
Regulation Of Mitotic Cell Cycle (66)
Traf6 Mediated Irf7 Activation (17)
Hiv Infection (167)
Host Interactions Of Hiv Factors (102)
Innate Immune System (192)
Bile Acid And Bile Salt Metabolism (22)
Signaling By Wnt (55)
Downstream Signaling Events Of B Cell Receptor Bcr (82)
Signaling By The B Cell Receptor Bcr (109)
Branched Chain Amino Acid Catabolism (16)
Signaling By Ils (98)
Nfkb Activation Through Fadd Rip1 Pathway Mediated By Caspase 8 And10 (11)
Phase Ii Conjugation (50)
Endosomal Vacuolar Pathway (5)
Immunoregulatory Interactions Between A Lymphoid And A Non Lymphoid Cell (46)
Srp Dependent Cotranslational Protein Targeting To Membrane (79)
Triglyceride Biosynthesis (37)
Collagen Formation (54)
Abacavir Transport And Metabolism (8)
Fatty Acyl Coa Biosynthesis (17)
Cytochrome P450 Arranged By Substrate Type (37)
Peptide Chain Elongation (57)
Abc Family Proteins Mediated Transport (27)
3 Utr Mediated Translational Regulation (72)
Traf6 Mediated Nfkb Activation (20)
Influenza Life Cycle (100)
Influenza Viral Rna Transcription And Replication (70)
Nonsense Mediated Decay Enhanced By The Exon Junction Complex (76)
Translation (111)
Synthesis Of Bile Acids And Bile Salts Via 7alpha Hydroxycholesterol (14)
Synthesis Of Bile Acids And Bile Salts (17)
Extracellular Matrix Organization (74)
Mhc Class Ii Antigen Presentation (79)
Phosphorylation Of Cd3 And Tcr Zeta Chains (12)
Translocation Of Zap 70 To Immunological Synapse (10)
Mitochondrial Fatty Acid Beta Oxidation (14)
P130cas Linkage To Mapk Signaling For Integrins (14)
Prefoldin Mediated Transfer Of Substrate To Cct Tric (22)
Costimulation By The Cd28 Family (56)
Respiratory Electron Transport Atp Synthesis By Chemiosmotic Coupling And Heat Production By Uncoupling Proteins (68)
Mitotic Prometaphase (78)
Extrinsic Pathway For Apoptosis (12)
Formation Of The Ternary Complex And Subsequently The 43s Complex (37)
Rip Mediated Nfkb Activation Via Dai (16)
Signaling By Bmp (19)
Generation Of Second Messenger Molecules (23)
Cytosolic Trna Aminoacylation (23)
Transmembrane Transport Of Small Molecules (303)
Integrin Alphaiib Beta3 Signaling (26)
Mrna Processing (139)
Formation Of Tubulin Folding Intermediates By Cct Tric (16)
Metabolism Of Proteins (346)
Mrna Splicing (97)
Ncam1 Interactions (30)
Pd1 Signaling (14)
Fatty Acid Triacylglycerol And Ketone Body Metabolism (160)
Respiratory Electron Transport (57)
Signal Regulatory Protein Sirp Family Interactions (10)
Platelet Aggregation Plug Formation (34)
Unwinding Of Dna (11)
Antigen Presentation Folding Assembly And Peptide Loading Of Class I Mhc (16)
Cholesterol Biosynthesis (20)
Pyruvate Metabolism And Citric Acid Tca Cycle (38)
Activation Of The Pre Replicative Complex (28)
Synthesis Of Bile Acids And Bile Salts Via 24 Hydroxycholesterol (10)
Androgen Biosynthesis (7)
Acyl Chain Remodelling Of Pi (9)
Alpha Linolenic Acid Ala Metabolism (11)
Egfr Downregulation (21)
Tcr Signaling (47)
Activation Of The Mrna Upon Binding Of The Cap Binding Complex And Eifs And Subsequent Binding To 43s (44)
Integrin Cell Surface Interactions (75)
Grb2 Sos Provides Linkage To Mapk Signaling For Intergrins (14)
Trna Aminoacylation (41)
Amine Derived Hormones (6)
Peroxisomal Lipid Metabolism (20)
Mrna Splicing Minor Pathway (38)
Unfolded Protein Response (70)
Slc Mediated Transmembrane Transport (182)
Formation Of Fibrin Clot Clotting Cascade (30)
Regulation Of Ifng Signaling (11)
Kinesins (21)
Il 2 Signaling (37)
Synthesis Of Very Long Chain Fatty Acyl Coas (13)
E2f Mediated Regulation Of Dna Replication (31)
Hemostasis (387)
Abortive Elongation Of Hiv1 Transcript In The Absence Of Tat (21)
G1 S Specific Transcription (15)
The Role Of Nef In Hiv1 Replication And Disease Pathogenesis (23)
Glutathione Conjugation (19)
Complement Cascade (25)
Toll Receptor Cascades (103)
Glucose Metabolism (57)
Platelet Activation Signaling And Aggregation (178)
G2 M Checkpoints (39)
Dna Strand Elongation (29)
AV 2 vs M
AV 5 vs M
AV 11 vs M
Jo 2 vs M
Jo 5 vs M
Jo 11 vs M
Jo vs AV (2)
Jo vs AV (5)
Jo vs AV (11)
Jo 5 vs 2
Jo 11 vs 5
Jo 11 vs 2
AV 5 vs 2
AV 11 vs 5
AV 11 vs 2

Fig S7c

Lymph nodes

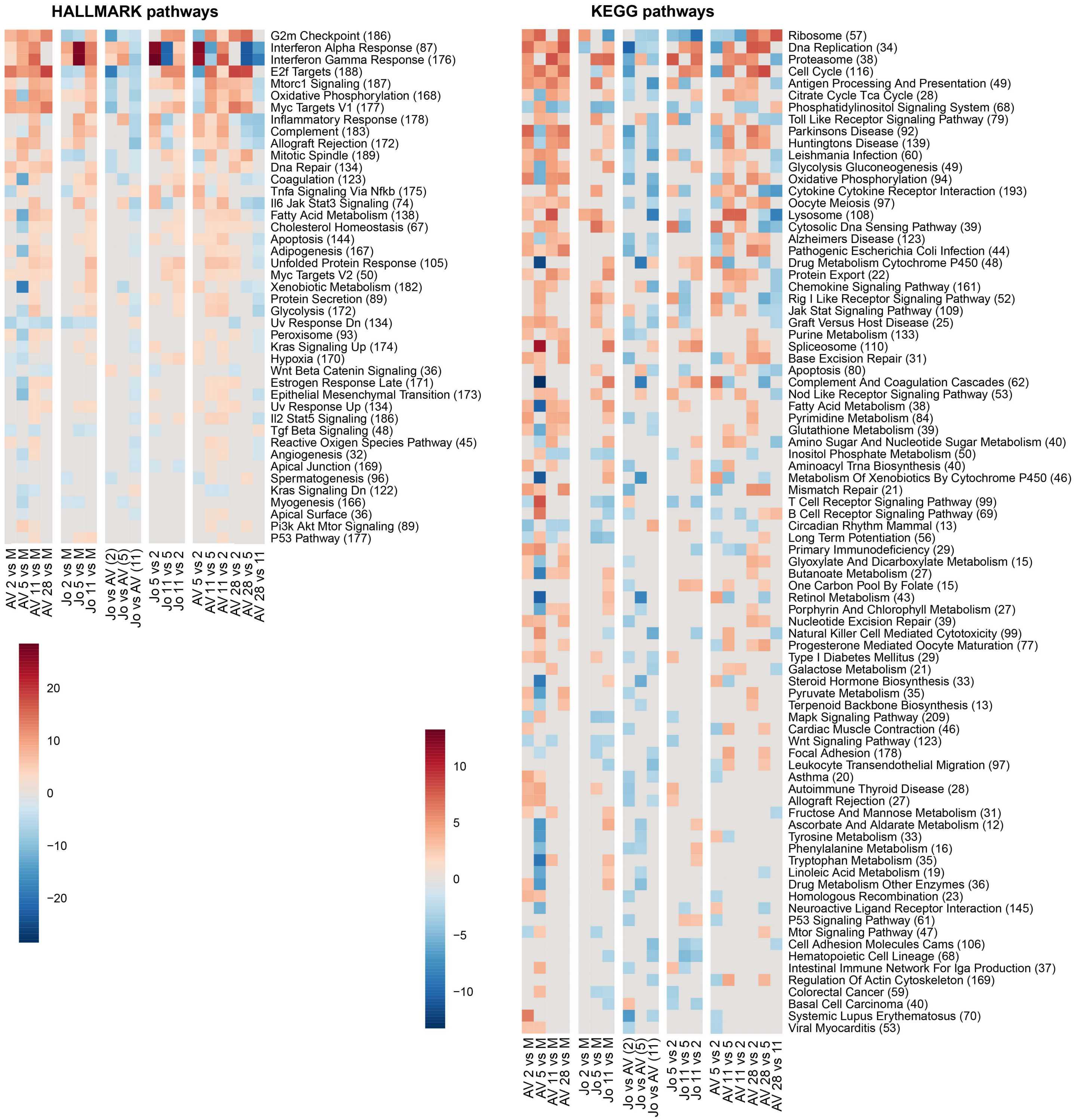

Fig S7d

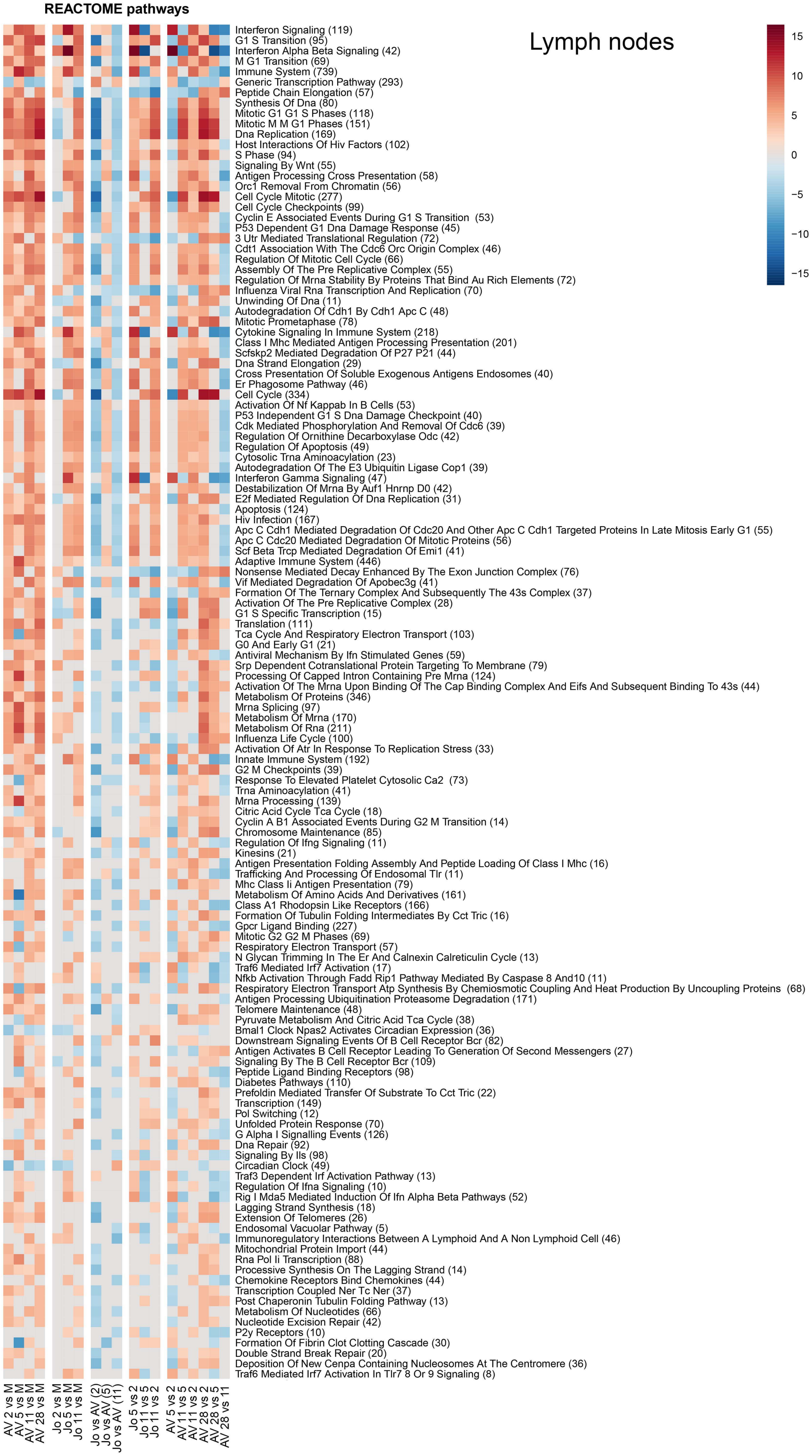

REACTOME pathways
Lymph nodes
15
10
5
0
−5
−10
−15
Interferon Signaling (119)
G1 S Transition (95)
Interferon Alpha Beta Signaling (42)
M G1 Transition (69)
Immune System (739)
Generic Transcription Pathway (293)
Peptide Chain Elongation (57)
Synthesis Of Dna (80)
Mitotic G1 G1 S Phases (118)
Mitotic M M G1 Phases (151)
Dna Replication (169)
Host Interactions Of Hiv Factors (102)
S Phase (94)
Signaling By Wnt (55)
Antigen Processing Cross Presentation (58)
Orc1 Removal From Chromatin (56)
Cell Cycle Mitotic (277)
Cell Cycle Checkpoints (99)
Cyclin E Associated Events During G1 S Transition (53)
P53 Dependent G1 Dna Damage Response (45)
3 Utr Mediated Translational Regulation (72)
Cdt1 Association With The Cdc6 Orc Origin Complex (46)
Regulation Of Mitotic Cell Cycle (66)
Assembly Of The Pre Replicative Complex (55)
Regulation Of Mrna Stability By Proteins That Bind Au Rich Elements (72)
Influenza Viral Rna Transcription And Replication (70)
Unwinding Of Dna (11)
Autodegradation Of Cdh1 By Cdh1 Apc C (48)
Mitotic Prometaphase (78)
Cytokine Signaling In Immune System (218)
Class I Mhc Mediated Antigen Processing Presentation (201)
Scfskp2 Mediated Degradation Of P27 P21 (44)
Dna Strand Elongation (29)
Cross Presentation Of Soluble Exogenous Antigens Endosomes (40)
Er Phagosome Pathway (46)
Cell Cycle (334)
Activation Of Nf Kappab In B Cells (53)
P53 Independent G1 S Dna Damage Checkpoint (40)
Cdk Mediated Phosphorylation And Removal Of Cdc6 (39)
Regulation Of Ornithine Decarboxylase Odc (42)
Regulation Of Apoptosis (49)
Cytosolic Trna Aminoacylation (23)
Autodegradation Of The E3 Ubiquitin Ligase Cop1 (39)
Interferon Gamma Signaling (47)
Destabilization Of Mrna By Auf1 Hnrnp D0 (42)
E2f Mediated Regulation Of Dna Replication (31)
Apoptosis (124)
Hiv Infection (167)
Apc C Cdh1 Mediated Degradation Of Cdc20 And Other Apc C Cdh1 Targeted Proteins In Late Mitosis Early G1 (55)
Apc C Cdc20 Mediated Degradation Of Mitotic Proteins (56)
Scf Beta Trcp Mediated Degradation Of Emi1 (41)
Adaptive Immune System (446)
Nonsense Mediated Decay Enhanced By The Exon Junction Complex (76)
Vif Mediated Degradation Of Apobec3g (41)
Formation Of The Ternary Complex And Subsequently The 43s Complex (37)
Activation Of The Pre Replicative Complex (28)
G1 S Specific Transcription (15)
Translation (111)
Tca Cycle And Respiratory Electron Transport (103)
G0 And Early G1 (21)
Antiviral Mechanism By Ifn Stimulated Genes (59)
Srp Dependent Cotranslational Protein Targeting To Membrane (79)
Processing Of Capped Intron Containing Pre Mrna (124)
Activation Of The Mrna Upon Binding Of The Cap Binding Complex And Eifs And Subsequent Binding To 43s (44)
Metabolism Of Proteins (346)
Mrna Splicing (97)
Metabolism Of Mrna (170)
Metabolism Of Rna (211)
Influenza Life Cycle (100)
Activation Of Atr In Response To Replication Stress (33)
Innate Immune System (192)
G2 M Checkpoints (39)
Response To Elevated Platelet Cytosolic Ca2 (73)
Trna Aminoacylation (41)
Mrna Processing (139)
Citric Acid Cycle Tca Cycle (18)
Cyclin A B1 Associated Events During G2 M Transition (14)
Chromosome Maintenance (85)
Regulation Of Ifng Signaling (11)
Kinesins (21)
Antigen Presentation Folding Assembly And Peptide Loading Of Class I Mhc (16)
Trafficking And Processing Of Endosomal Tlr (11)
Mhc Class Ii Antigen Presentation (79)
Metabolism Of Amino Acids And Derivatives (161)
Class A1 Rhodopsin Like Receptors (166)
Formation Of Tubulin Folding Intermediates By Cct Tric (16)
Gpcr Ligand Binding (227)
Mitotic G2 G2 M Phases (69)
Respiratory Electron Transport (57)
N Glycan Trimming In The Er And Calnexin Calreticulin Cycle (13)
Traf6 Mediated Irf7 Activation (17)
Nfkb Activation Through Fadd Rip1 Pathway Mediated By Caspase 8 And10 (11)
Respiratory Electron Transport Atp Synthesis By Chemiosmotic Coupling And Heat Production By Uncoupling Proteins (68)
Antigen Processing Ubiquitination Proteasome Degradation (171)
Telomere Maintenance (48)
Pyruvate Metabolism And Citric Acid Tca Cycle (38)
Bmal1 Clock Npas2 Activates Circadian Expression (36)
Downstream Signaling Events Of B Cell Receptor Bcr (82)
Antigen Activates B Cell Receptor Leading To Generation Of Second Messengers (27)
Signaling By The B Cell Receptor Bcr (109)
Peptide Ligand Binding Receptors (98)
Diabetes Pathways (110)
Prefoldin Mediated Transfer Of Substrate To Cct Tric (22)
Transcription (149)
Pol Switching (12)
Unfolded Protein Response (70)
G Alpha I Signalling Events (126)
Dna Repair (92)
Signaling By Ils (98)
Circadian Clock (49)
Traf3 Dependent Irf Activation Pathway (13)
Regulation Of Ifna Signaling (10)
Rig I Mda5 Mediated Induction Of Ifn Alpha Beta Pathways (52)
Lagging Strand Synthesis (18)
Extension Of Telomeres (26)
Endosomal Vacuolar Pathway (5)
Immunoregulatory Interactions Between A Lymphoid And A Non Lymphoid Cell (46)
Mitochondrial Protein Import (44)
Rna Pol Ii Transcription (88)
Processive Synthesis On The Lagging Strand (14)
Chemokine Receptors Bind Chemokines (44)
Transcription Coupled Ner Tc Ner (37)
Post Chaperonin Tubulin Folding Pathway (13)
Metabolism Of Nucleotides (66)
Nucleotide Excision Repair (42)
P2y Receptors (10)
Formation Of Fibrin Clot Clotting Cascade (30)
Double Strand Break Repair (20)
Deposition Of New Cenpa Containing Nucleosomes At The Centromere (36)
Traf6 Mediated Irf7 Activation In Tlr7 8 Or 9 Signaling (8)
AV 2 vs M
AV 5 vs M
AV 11 vs M
AV 28 vs M
Jo 2 vs M
Jo 5 vs M
Jo 11 vs M
Jo vs AV (2)
Jo vs AV (5)
Jo vs AV (11)
Jo 5 vs 2
Jo 11 vs 5
Jo 11 vs 2
AV 5 vs 2
AV 11 vs 5
AV 11 vs 2
AV 28 vs 2
AV 28 vs 5
AV 28 vs 11

Fig S7e

Lymph nodes

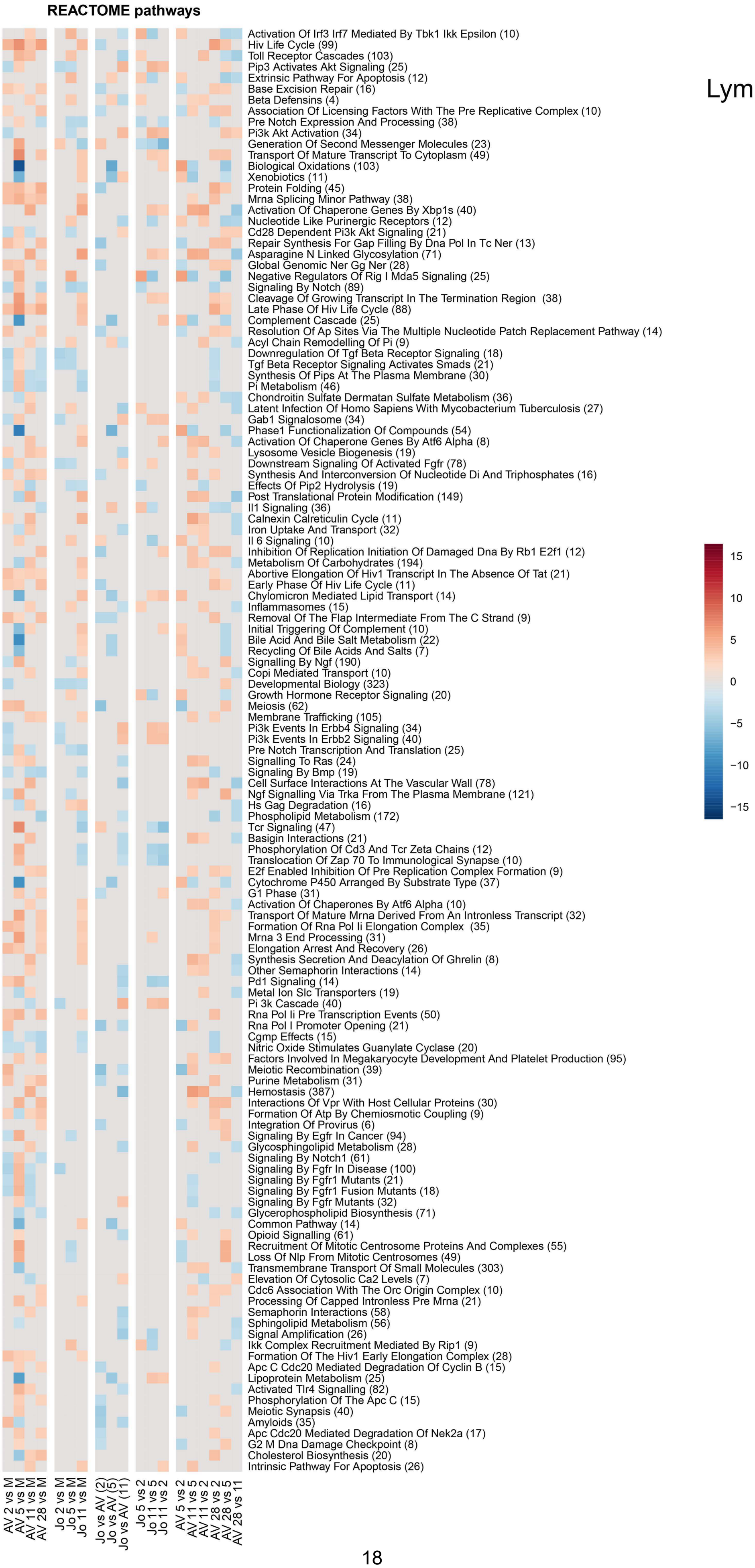

Fig S7f

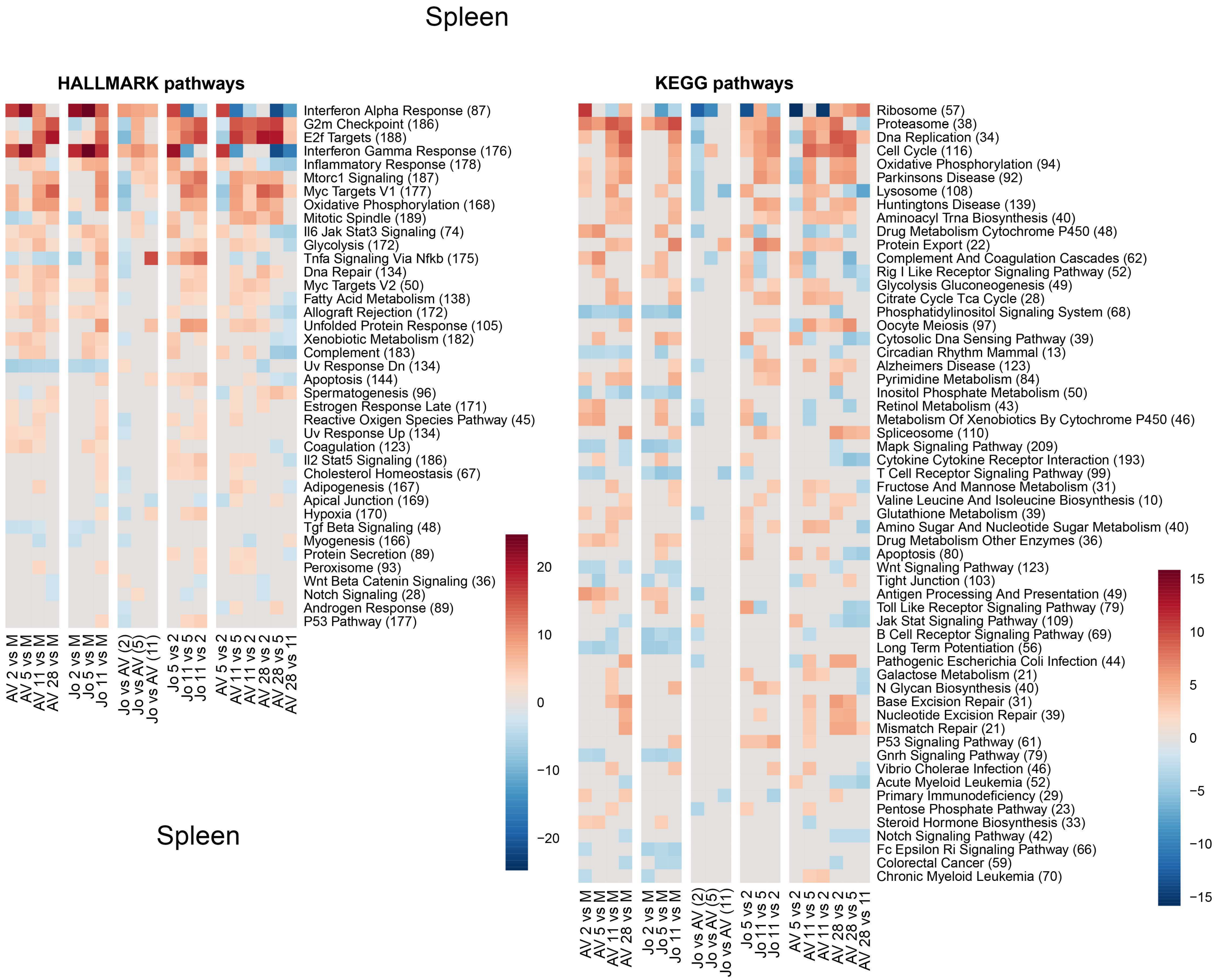

Spleen
HALLMARK pathways
KEGG pathways
Interferon Alpha Response (87)
G2m Checkpoint (186)
E2f Targets (188)
Interferon Gamma Response (176)
Inflammatory Response (178)
Mtorc1 Signaling (187)
Myc Targets V1 (177)
Oxidative Phosphorylation (168)
Mitotic Spindle (189)
Il6 Jak Stat3 Signaling (74)
Glycolysis (172)
Tnfa Signaling Via Nfkb (175)
Dna Repair (134)
Myc Targets V2 (50)
Fatty Acid Metabolism (138)
Allograft Rejection (172)
Unfolded Protein Response (105)
Xenobiotic Metabolism (182)
Complement (183)
Uv Response Dn (134)
Apoptosis (144)
Spermatogenesis (96)
Estrogen Response Late (171)
Reactive Oxigen Species Pathway (45)
Uv Response Up (134)
Coagulation (123)
Il2 Stat5 Signaling (186)
Cholesterol Homeostasis (67)
Adipogenesis (167)
Apical Junction (169)
Hypoxia (170)
Tgf Beta Signaling (48)
Myogenesis (166)
Protein Secretion (89)
Peroxisome (93)
Wnt Beta Catenin Signaling (36)
Notch Signaling (28)
Androgen Response (89)
P53 Pathway (177)
AV 2 vs M
AV 5 vs M
AV 11 vs M
AV 28 vs M
Jo 2 vs M
Jo 5 vs M
Jo 11 vs M
Jo vs AV (2)
Jo vs AV (5)
Jo vs AV (11)
Jo 5 vs 2
Jo 11 vs 5
Jo 11 vs 2
AV 5 vs 2
AV 11 vs 5
AV 11 vs 2
AV 28 vs 2
AV 28 vs 5
AV 28 vs 11
20
10
0
−10
−20
Spleen
Ribosome (57)
Proteasome (38)
Dna Replication (34)
Cell Cycle (116)
Oxidative Phosphorylation (94)
Parkinsons Disease (92)
Lysosome (108)
Huntingtons Disease (139)
Aminoacyl Trna Biosynthesis (40)
Drug Metabolism Cytochrome P450 (48)
Protein Export (22)
Complement And Coagulation Cascades (62)
Rig I Like Receptor Signaling Pathway (52)
Glycolysis Gluconeogenesis (49)
Citrate Cycle Tca Cycle (28)
Phosphatidylinositol Signaling System (68)
Oocyte Meiosis (97)
Cytosolic Dna Sensing Pathway (39)
Circadian Rhythm Mammal (13)
Alzheimers Disease (123)
Pyrimidine Metabolism (84)
Inositol Phosphate Metabolism (50)
Retinol Metabolism (43)
Metabolism Of Xenobiotics By Cytochrome P450 (46)
Spliceosome (110)
Mapk Signaling Pathway (209)
Cytokine Cytokine Receptor Interaction (193)
T Cell Receptor Signaling Pathway (99)
Fructose And Mannose Metabolism (31)
Valine Leucine And Isoleucine Biosynthesis (10)
Glutathione Metabolism (39)
Amino Sugar And Nucleotide Sugar Metabolism (40)
Drug Metabolism Other Enzymes (36)
Apoptosis (80)
Wnt Signaling Pathway (123)
Tight Junction (103)
Antigen Processing And Presentation (49)
Toll Like Receptor Signaling Pathway (79)
Jak Stat Signaling Pathway (109)
B Cell Receptor Signaling Pathway (69)
Long Term Potentiation (56)
Pathogenic Escherichia Coli Infection (44)
Galactose Metabolism (21)
N Glycan Biosynthesis (40)
Base Excision Repair (31)
Nucleotide Excision Repair (39)
Mismatch Repair (21)
P53 Signaling Pathway (61)
Gnrh Signaling Pathway (79)
Vibrio Cholerae Infection (46)
Acute Myeloid Leukemia (52)
Primary Immunodeficiency (29)
Pentose Phosphate Pathway (23)
Steroid Hormone Biosynthesis (33)
Notch Signaling Pathway (42)
Fc Epsilon Ri Signaling Pathway (66)
Colorectal Cancer (59)
Chronic Myeloid Leukemia (70)
AV 2 vs M
AV 5 vs M
AV 11 vs M
AV 28 vs M
Jo 2 vs M
Jo 5 vs M
Jo 11 vs M
Jo vs AV (2)
Jo vs AV (5)
Jo vs AV (11)
Jo 5 vs 2
Jo 11 vs 5
Jo 11 vs 2
AV 5 vs 2
AV 11 vs 5
AV 11 vs 2
AV 28 vs 2
AV 28 vs 5
AV 28 vs 11
15
10
5
0
−5
−10
−15

Fig S7g

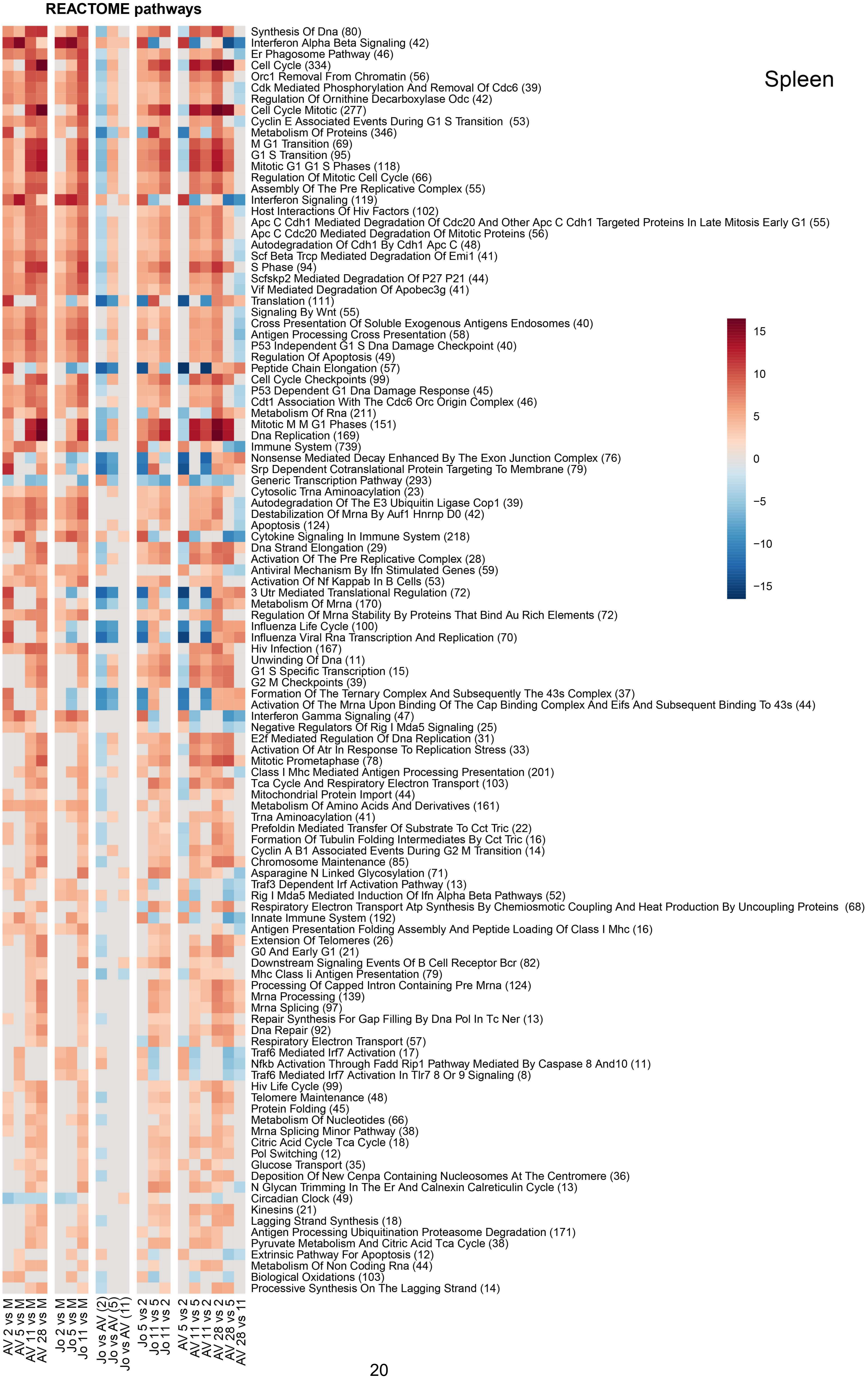

REACTOME pathways
Spleen
Synthesis Of Dna (80)
Interferon Alpha Beta Signaling (42)
Er Phagosome Pathway (46)
Cell Cycle (334)
Orc1 Removal From Chromatin (56)
Cdk Mediated Phosphorylation And Removal Of Cdc6 (39)
Regulation Of Ornithine Decarboxylase Odc (42)
Cell Cycle Mitotic (277)
Cyclin E Associated Events During G1 S Transition (53)
Metabolism Of Proteins (346)
M G1 Transition (69)
G1 S Transition (95)
Mitotic G1 G1 S Phases (118)
Regulation Of Mitotic Cell Cycle (66)
Assembly Of The Pre Replicative Complex (55)
Interferon Signaling (119)
Host Interactions Of Hiv Factors (102)
Apc C Cdh1 Mediated Degradation Of Cdc20 And Other Apc C Cdh1 Targeted Proteins In Late Mitosis Early G1 (55)
Apc C Cdc20 Mediated Degradation Of Mitotic Proteins (56)
Autodegradation Of Cdh1 By Cdh1 Apc C (48)
Scf Beta Trcp Mediated Degradation Of Emi1 (41)
S Phase (94)
Scfskp2 Mediated Degradation Of P27 P21 (44)
Vif Mediated Degradation Of Apobec3g (41)
Translation (111)
Signaling By Wnt (55)
Cross Presentation Of Soluble Exogenous Antigens Endosomes (40)
Antigen Processing Cross Presentation (58)
P53 Independent G1 S Dna Damage Checkpoint (40)
Regulation Of Apoptosis (49)
Peptide Chain Elongation (57)
Cell Cycle Checkpoints (99)
P53 Dependent G1 Dna Damage Response (45)
Cdt1 Association With The Cdc6 Orc Origin Complex (46)
Metabolism Of Rna (211)
Mitotic M M G1 Phases (151)
Dna Replication (169)
Immune System (739)
Nonsense Mediated Decay Enhanced By The Exon Junction Complex (76)
Srp Dependent Cotranslational Protein Targeting To Membrane (79)
Generic Transcription Pathway (293)
Cytosolic Trna Aminoacylation (23)
Autodegradation Of The E3 Ubiquitin Ligase Cop1 (39)
Destabilization Of Mrna By Auf1 Hnrnp D0 (42)
Apoptosis (124)
Cytokine Signaling In Immune System (218)
Dna Strand Elongation (29)
Activation Of The Pre Replicative Complex (28)
Antiviral Mechanism By Ifn Stimulated Genes (59)
Activation Of Nf Kappab In B Cells (53)
3 Utr Mediated Translational Regulation (72)
Metabolism Of Mrna (170)
Regulation Of Mrna Stability By Proteins That Bind Au Rich Elements (72)
Influenza Life Cycle (100)
Influenza Viral Rna Transcription And Replication (70)
Hiv Infection (167)
Unwinding Of Dna (11)
G1 S Specific Transcription (15)
G2 M Checkpoints (39)
Formation Of The Ternary Complex And Subsequently The 43s Complex (37)
Activation Of The Mrna Upon Binding Of The Cap Binding Complex And Eifs And Subsequent Binding To 43s (44)
Interferon Gamma Signaling (47)
Negative Regulators Of Rig I Mda5 Signaling (25)
E2f Mediated Regulation Of Dna Replication (31)
Activation Of Atr In Response To Replication Stress (33)
Mitotic Prometaphase (78)
Class I Mhc Mediated Antigen Processing Presentation (201)
Tca Cycle And Respiratory Electron Transport (103)
Mitochondrial Protein Import (44)
Metabolism Of Amino Acids And Derivatives (161)
Trna Aminoacylation (41)
Prefoldin Mediated Transfer Of Substrate To Cct Tric (22)
Formation Of Tubulin Folding Intermediates By Cct Tric (16)
Cyclin A B1 Associated Events During G2 M Transition (14)
Chromosome Maintenance (85)
Asparagine N Linked Glycosylation (71)
Traf3 Dependent Irf Activation Pathway (13)
Rig I Mda5 Mediated Induction Of Ifn Alpha Beta Pathways (52)
Respiratory Electron Transport Atp Synthesis By Chemiosmotic Coupling And Heat Production By Uncoupling Proteins (68)
Innate Immune System (192)
Antigen Presentation Folding Assembly And Peptide Loading Of Class I Mhc (16)
Extension Of Telomeres (26)
G0 And Early G1 (21)
Downstream Signaling Events Of B Cell Receptor Bcr (82)
Mhc Class Ii Antigen Presentation (79)
Processing Of Capped Intron Containing Pre Mrna (124)
Mrna Processing (139)
Mrna Splicing (97)
Repair Synthesis For Gap Filling By Dna Pol In Tc Ner (13)
Dna Repair (92)
Respiratory Electron Transport (57)
Traf6 Mediated Irf7 Activation (17)
Nfkb Activation Through Fadd Rip1 Pathway Mediated By Caspase 8 And10 (11)
Traf6 Mediated Irf7 Activation In Tlr7 8 Or 9 Signaling (8)
Hiv Life Cycle (99)
Telomere Maintenance (48)
Protein Folding (45)
Metabolism Of Nucleotides (66)
Mrna Splicing Minor Pathway (38)
Citric Acid Cycle Tca Cycle (18)
Pol Switching (12)
Glucose Transport (35)
Deposition Of New Cenpa Containing Nucleosomes At The Centromere (36)
N Glycan Trimming In The Er And Calnexin Calreticulin Cycle (13)
Circadian Clock (49)
Kinesins (21)
Lagging Strand Synthesis (18)
Antigen Processing Ubiquitination Proteasome Degradation (171)
Pyruvate Metabolism And Citric Acid Tca Cycle (38)
Extrinsic Pathway For Apoptosis (12)
Metabolism Of Non Coding Rna (44)
Biological Oxidations (103)
Processive Synthesis On The Lagging Strand (14)
15
10
5
0
−5
−10
−15
AV 2 vs M
AV 5 vs M
AV 11 vs M
AV 28 vs M
Jo 2 vs M
Jo 5 vs M
Jo 11 vs M
Jo vs AV (2)
Jo vs AV (5)
Jo vs AV (11)
Jo 5 vs 2
Jo 11 vs 5
Jo 11 vs 2
AV 5 vs 2
AV 11 vs 5
AV 11 vs 2
AV 28 vs 2
AV 28 vs 5
AV 28 vs 11

Fig S7h

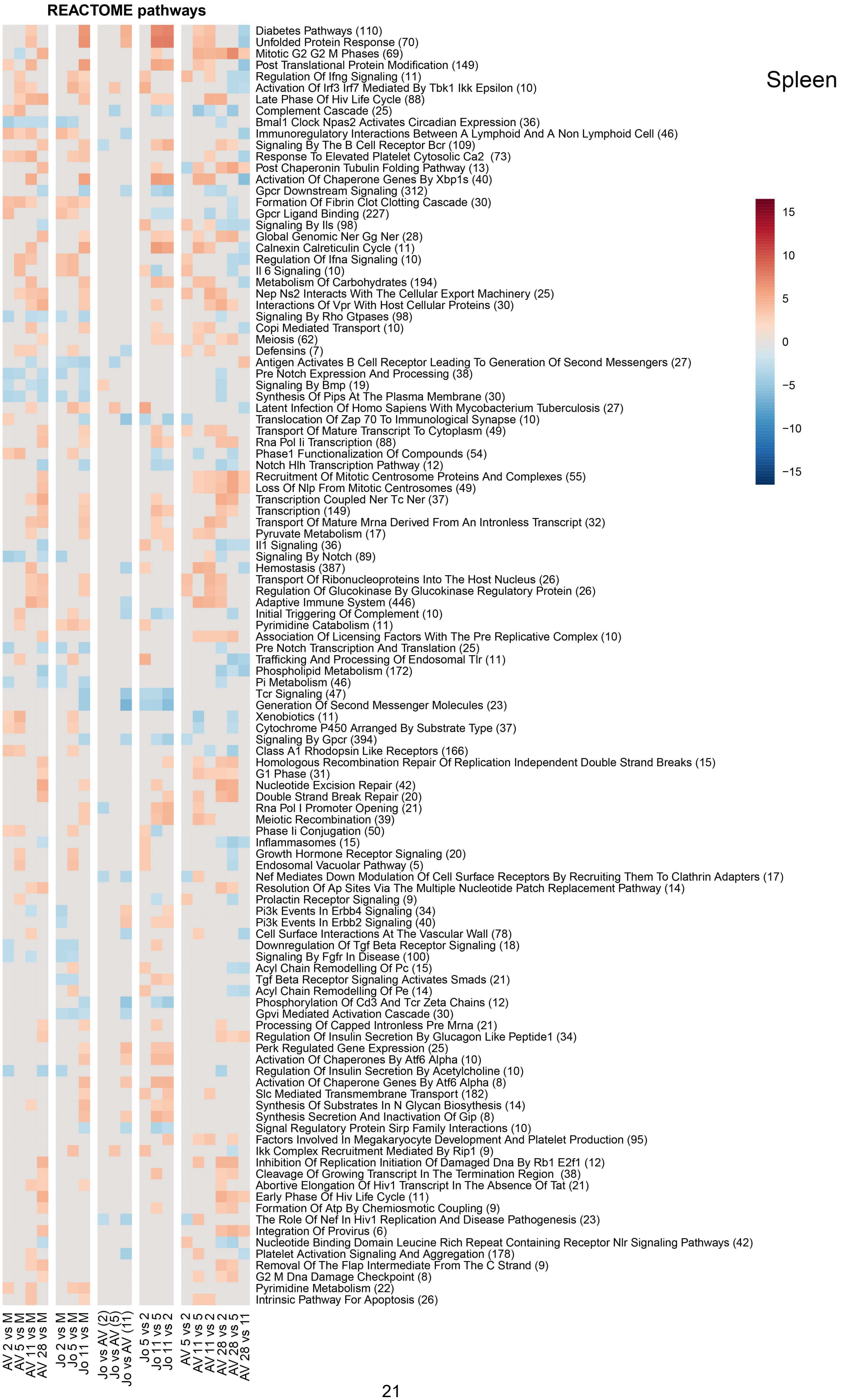

REACTOME pathways
Spleen
Diabetes Pathways (110)
Unfolded Protein Response (70)
Mitotic G2 G2 M Phases (69)
Post Translational Protein Modification (149)
Regulation Of Ifng Signaling (11)
Activation Of Irf3 Irf7 Mediated By Tbk1 Ikk Epsilon (10)
Late Phase Of Hiv Life Cycle (88)
Complement Cascade (25)
Bmal1 Clock Npas2 Activates Circadian Expression (36)
Immunoregulatory Interactions Between A Lymphoid And A Non Lymphoid Cell (46)
Signaling By The B Cell Receptor Bcr (109)
Response To Elevated Platelet Cytosolic Ca2 (73)
Post Chaperonin Tubulin Folding Pathway (13)
Activation Of Chaperone Genes By Xbp1s (40)
Gpcr Downstream Signaling (312)
Formation Of Fibrin Clot Clotting Cascade (30)
Gpcr Ligand Binding (227)
Signaling By Ils (98)
Global Genomic Ner Gg Ner (28)
Calnexin Calreticulin Cycle (11)
Regulation Of Ifna Signaling (10)
Il 6 Signaling (10)
Metabolism Of Carbohydrates (194)
Nep Ns2 Interacts With The Cellular Export Machinery (25)
Interactions Of Vpr With Host Cellular Proteins (30)
Signaling By Rho Gtpases (98)
Copi Mediated Transport (10)
Meiosis (62)
Defensins (7)
Antigen Activates B Cell Receptor Leading To Generation Of Second Messengers (27)
Pre Notch Expression And Processing (38)
Signaling By Bmp (19)
Synthesis Of Pips At The Plasma Membrane (30)
Latent Infection Of Homo Sapiens With Mycobacterium Tuberculosis (27)
Translocation Of Zap 70 To Immunological Synapse (10)
Transport Of Mature Transcript To Cytoplasm (49)
Rna Pol Ii Transcription (88)
Phase1 Functionalization Of Compounds (54)
Notch Hlh Transcription Pathway (12)
Recruitment Of Mitotic Centrosome Proteins And Complexes (55)
Loss Of Nlp From Mitotic Centrosomes (49)
Transcription Coupled Ner Tc Ner (37)
Transcription (149)
Transport Of Mature Mrna Derived From An Intronless Transcript (32)
Pyruvate Metabolism (17)
Il1 Signaling (36)
Signaling By Notch (89)
Hemostasis (387)
Transport Of Ribonucleoproteins Into The Host Nucleus (26)
Regulation Of Glucokinase By Glucokinase Regulatory Protein (26)
Adaptive Immune System (446)
Initial Triggering Of Complement (10)
Pyrimidine Catabolism (11)
Association Of Licensing Factors With The Pre Replicative Complex (10)
Pre Notch Transcription And Translation (25)
Trafficking And Processing Of Endosomal Tlr (11)
Phospholipid Metabolism (172)
Pi Metabolism (46)
Tcr Signaling (47)
Generation Of Second Messenger Molecules (23)
Xenobiotics (11)
Cytochrome P450 Arranged By Substrate Type (37)
Signaling By Gpcr (394)
Class A1 Rhodopsin Like Receptors (166)
Homologous Recombination Repair Of Replication Independent Double Strand Breaks (15)
G1 Phase (31)
Nucleotide Excision Repair (42)
Double Strand Break Repair (20)
Rna Pol I Promoter Opening (21)
Meiotic Recombination (39)
Phase Ii Conjugation (50)
Inflammasomes (15)
Growth Hormone Receptor Signaling (20)
Endosomal Vacuolar Pathway (5)
Nef Mediates Down Modulation Of Cell Surface Receptors By Recruiting Them To Clathrin Adapters (17)
Resolution Of Ap Sites Via The Multiple Nucleotide Patch Replacement Pathway (14)
Prolactin Receptor Signaling (9)
Pi3k Events In Erbb4 Signaling (34)
Pi3k Events In Erbb2 Signaling (40)
Cell Surface Interactions At The Vascular Wall (78)
Downregulation Of Tgf Beta Receptor Signaling (18)
Signaling By Fgfr In Disease (100)
Acyl Chain Remodelling Of Pc (15)
Tgf Beta Receptor Signaling Activates Smads (21)
Acyl Chain Remodelling Of Pe (14)
Phosphorylation Of Cd3 And Tcr Zeta Chains (12)
Gpvi Mediated Activation Cascade (30)
Processing Of Capped Intronless Pre Mrna (21)
Regulation Of Insulin Secretion By Glucagon Like Peptide1 (34)
Perk Regulated Gene Expression (25)
Activation Of Chaperones By Atf6 Alpha (10)
Regulation Of Insulin Secretion By Acetylcholine (10)
Activation Of Chaperone Genes By Atf6 Alpha (8)
Slc Mediated Transmembrane Transport (182)
Synthesis Of Substrates In N Glycan Biosythesis (14)
Synthesis Secretion And Inactivation Of Gip (8)
Signal Regulatory Protein Sirp Family Interactions (10)
Factors Involved In Megakaryocyte Development And Platelet Production (95)
Ikk Complex Recruitment Mediated By Rip1 (9)
Inhibition Of Replication Initiation Of Damaged Dna By Rb1 E2f1 (12)
Cleavage Of Growing Transcript In The Termination Region (38)
Abortive Elongation Of Hiv1 Transcript In The Absence Of Tat (21)
Early Phase Of Hiv Life Cycle (11)
Formation Of Atp By Chemiosmotic Coupling (9)
The Role Of Nef In Hiv1 Replication And Disease Pathogenesis (23)
Integration Of Provirus (6)
Nucleotide Binding Domain Leucine Rich Repeat Containing Receptor Nlr Signaling Pathways (42)
Platelet Activation Signaling And Aggregation (178)
Removal Of The Flap Intermediate From The C Strand (9)
G2 M Dna Damage Checkpoint (8)
Pyrimidine Metabolism (22)
Intrinsic Pathway For Apoptosis (26)
15
10
5
0
−5
−10
−15
AV 2 vs M
AV 5 vs M
AV 11 vs M
AV 28 vs M
Jo 2 vs M
Jo 5 vs M
Jo 11 vs M
Jo vs AV (2)
Jo vs AV (5)
Jo vs AV (11)
Jo 5 vs 2
Jo 11 vs 5
Jo 11 vs 2
AV 5 vs 2
AV 11 vs 5
AV 11 vs 2
AV 28 vs 2
AV 28 vs 5
AV 28 vs 11

Fig S7. Heatmaps of the most significant pathways by gene-set analysis in the organs. The same approach as the one used in Fig S6 was applied to liver (Fig. S7a and b), MLN (Fig. S7c-e), and spleen (Fig. S7f-h) samples obtained 2, 5, and 11 DPI from LASV-infected NHPs and 28 DPI from AV and mock-infected animals.
