# Supplementary material for: Systemic viral spreading and defective host responses are associated with fatal Lassa fever in macaques
Source: Commun Biol. 2021 Jan 4;4:27. doi: 10.1038/s42003-020-01543-7 (PMC7782745; doi:10.1038/s42003-020-01543-7)
Supplement: Supplementary file 3 — Description of Supplementary Files [file 42003_2020_1543_MOESM3_ESM.pdf]

Description of Additional Supplementary Files

**File name:** Supplementary Data 1, Supplementary Data 2

**Description:** Raw data for all main figures.
