# Supplementary material for: Systemic viral spreading and defective host responses are associated with fatal Lassa fever in macaques
Source: Commun Biol. 2021 Jan 4;4:27. doi: 10.1038/s42003-020-01543-7 (PMC7782745; doi:10.1038/s42003-020-01543-7)
Supplement: Supplementary file 6 — Reporting Summary [file 42003_2020_1543_MOESM6_ESM.pdf]

nature research

Corresponding author(s): Sylvain Baize

Last updated by author(s): Oct 6, 2020

Reporting Summary

Nature Research wishes to improve the reproducibility of the work that we publish. This form provides structure for consistency and transparency in reporting. For further information on Nature Research policies, see our Editorial Policies and the Editorial Policy Checklist.

Statistics

For all statistical analyses, confirm that the following items are present in the figure legend, table legend, main text, or Methods section.

n/a	Confirmed	
☐	☒	The exact sample size (*n*) for each experimental group/condition, given as a discrete number and unit of measurement
☐	☒	A statement on whether measurements were taken from distinct samples or whether the same sample was measured repeatedly
☐	☒	The statistical test(s) used AND whether they are one- or two-sided<br>*Only common tests should be described solely by name; describe more complex techniques in the Methods section.*
☒	☐	A description of all covariates tested
☐	☒	A description of any assumptions or corrections, such as tests of normality and adjustment for multiple comparisons
☒	☐	A full description of the statistical parameters including central tendency (e.g. means) or other basic estimates (e.g. regression coefficient) AND variation (e.g. standard deviation) or associated estimates of uncertainty (e.g. confidence intervals)
☐	☒	For null hypothesis testing, the test statistic (e.g. *F*, *t*, *r*) with confidence intervals, effect sizes, degrees of freedom and *P* value noted<br>*Give P values as exact values whenever suitable.*
☒	☐	For Bayesian analysis, information on the choice of priors and Markov chain Monte Carlo settings
☒	☐	For hierarchical and complex designs, identification of the appropriate level for tests and full reporting of outcomes
☒	☐	Estimates of effect sizes (e.g. Cohen's *d*, Pearson's *r*), indicating how they were calculated

Our web collection on statistics for biologists contains articles on many of the points above.

Software and code

Policy information about availability of computer code

Data collection	*Provide a description of all commercial, open source and custom code used to collect the data in this study, specifying the version used OR state that no software was used.*
Data analysis	*Provide a description of all commercial, open source and custom code used to analyse the data in this study, specifying the version used OR state that no software was used.*

For manuscripts utilizing custom algorithms or software that are central to the research but not yet described in published literature, software must be made available to editors and reviewers. We strongly encourage code deposition in a community repository (e.g. GitHub). See the Nature Research guidelines for submitting code & software for further information.

Data

Policy information about availability of data

All manuscripts must include a data availability statement. This statement should provide the following information, where applicable:

- Accession codes, unique identifiers, or web links for publicly available datasets
- A list of figures that have associated raw data
- A description of any restrictions on data availability

The transcriptomic datasets generated during and/or analysed during the current study are not publicly available due to further investigation currently in progress but are available from the corresponding author on reasonable request

Field-specific reporting

Please select the one below that is the best fit for your research. If you are not sure, read the appropriate sections before making your selection.

- [x] Life sciences
- [ ] Behavioural & social sciences
- [ ] Ecological, evolutionary & environmental sciences

For a reference copy of the document with all sections, see nature.com/documents/nr-reporting-summary-flat.pdf

Life sciences study design

All studies must disclose on these points even when the disclosure is negative.

Sample size	The sample size was defined according to the 3R rules and to the need for statistical analysis.<br>For whole disease course follow up, non infected , AV- and Josiah-infected monkey numbers were 3, 4, and 6, respectively.<br>For selected time-point analysis, 3 animals were included in each group
Data exclusions	No data exclusion was performed
Replication	N/A
Randomization	Animals were randomly placed into each group
Blinding	It was not possible to perform blind experiments for containment reasons, experiment design, and administrative regulation about selected agents

Reporting for specific materials, systems and methods

We require information from authors about some types of materials, experimental systems and methods used in many studies. Here, indicate whether each material, system or method listed is relevant to your study. If you are not sure if a list item applies to your research, read the appropriate section before selecting a response.

Materials & experimental systems

n/a	Involved in the study	
[ ]	[x]	Antibodies
[ ]	[x]	Eukaryotic cell lines
[x]	[ ]	Palaeontology and archaeology
[ ]	[x]	Animals and other organisms
[x]	[ ]	Human research participants
[x]	[ ]	Clinical data
[x]	[ ]	Dual use research of concern

Methods

n/a	Involved in the study	
[x]	[ ]	ChIP-seq
[ ]	[x]	Flow cytometry
[x]	[ ]	MRI-based neuroimaging

Antibodies

Antibodies used	BD Biosciences: CD3 BV510 (560770), CD3 BV421 (560351), CD3 APCH7 (557757), CD4 APC (551980), CD4 Alexa700 (560836), CD8 APCH7 (641400), CD10 PE (557143), CD20 PC7 (560735), CD20 APC (560900), CD27 FITC (555440), CD27 APC (561400), CD28 PECF594 (562296), CD45RA PC7 (561216), CD69 PERCP5.5 (560738), CD80 BV510 (563084), CD95 APC (558814), Ki67 PC7 (561283), GrzB Alexa700 (560213), CD56 V450 (560360), CD20 PECF594 (562295), CD20 BV510 (563067), CD27 Alexa700 (560611), CXCR3 BV421 (562558), CD107a FITC (555800).<br><br>Miltenyi Bitotech: CD86 Vioblue (130-100-100), CD1c FITC (130-090-507), HLADR APCVio700 (130-104-200), CD14 APC (130-113-143), NKp80 FITC (130-094-843), KKG2d PE (130-123-709)<br><br>BioLegend: CD279 FITC (329904), CD40 PE (334308), CD16 Alexa700 (302026)<br>Mabtech: Perforin FITC (3465-7)<br>Stemcell : CD38 FITC (60131FI)
Validation	Please see the Manufacturer's instructions for each antibody for further information about the clones used and the validation.

Eukaryotic cell lines

Policy information about cell lines

Cell line source(s)	Vero E6

Authentication	Cell line has been obtained with ATCC
Mycoplasma contamination	The cell line was tested for mycoplasma contamination with a negative result
Commonly misidentified lines (See ICLAC register)	*Name any commonly misidentified cell lines used in the study and provide a rationale for their use.*

Animals and other organisms

Policy information about studies involving animals; ARRIVE guidelines recommended for reporting animal research

Laboratory animals	28 cynomolgus Monkey (Malaysia) : 12[F]; 16[M] between 2 and 3 years.
Wild animals	N/A
Field-collected samples	N/A
Ethics oversight	French Comité Régional d'Ethique pour l'Expérimentation Animale Rhône Alpes (file number 2015062410456662, CECAPP, UMS3444/US8, Lyon, France)

Note that full information on the approval of the study protocol must also be provided in the manuscript.

Flow Cytometry

Plots

Confirm that:

- [ ] The axis labels state the marker and fluorochrome used (e.g. CD4-FITC).
- [x] The axis scales are clearly visible. Include numbers along axes only for bottom left plot of group (a 'group' is an analysis of identical markers).
- [x] All plots are contour plots with outliers or pseudocolor plots.
- [x] A numerical value for number of cells or percentage (with statistics) is provided.

Methodology

Sample preparation	Quantification of blood cells populations, proliferation and cytotoxic activity during the course of the disease was measured from 50µL of fresh whole blood. For surface staining only, cells were fixed after lysing of the red blood cells. When intracellular staining was used, red blood cells were lysed and white cells were stained with surface antibodies, permeabilized, and stained using antibodies before fixation in PBS with 1% paraformaldehyde (PFA).<br>Cells from spleen, mesenteric and inguinal lymph nodes were manually crushed from organs and filtered using a 70 µm cell strainer. Red blood cells were then lysed and cells from organs suspended in fresh medium. Cells were then directly stained for intracellular and extracellular markers using the same protocol and reagents described above.
Instrument	10-color Gallios cytometer (Beckman Coulter)
Software	Data were analyzed using Kaluza software 2.1 (Beckman Coulter).
Cell population abundance	N/A
Gating strategy	Leucocytes were first selected by gating the singlet cells using a FS Int/FS TOF dot plot. Lymphocytes were then subselected from singlets using a CD45/SS Int dot plot. Subpopulations of lymphocytes were then selected using CD3, CD4, CD8, and CD20 antibodies

- [ ] Tick this box to confirm that a figure exemplifying the gating strategy is provided in the Supplementary Information.
